# New anti-diabetic agents for the treatment of non-alcoholic fatty liver disease: a systematic review and network meta-analysis of randomized controlled trials

**DOI:** 10.3389/fendo.2023.1182037

**Published:** 2023-06-27

**Authors:** Tanawan Kongmalai, Varalak Srinonprasert, Thunyarat Anothaisintawee, Pinkawas Kongmalai, Gareth McKay, John Attia, Ammarin Thakkinstian

**Affiliations:** ^1^ Division of Endocrinology and Metabolism, Department of Medicine, Faculty of Medicine, Siriraj Hospital, Mahidol University, Bangkok, Thailand; ^2^ Siriraj Health Policy Unit, Faculty of Medicine, Siriraj Hospital, Mahidol University, Bangkok, Thailand; ^3^ Division of Geriatric Medicine, Department of Medicine, Faculty of Medicine, Siriraj Hospital, Mahidol University, Bangkok, Thailand; ^4^ Mahidol University Health Technology Assessment Graduate Program, Mahidol University, Bangkok, Thailand; ^5^ Department of Clinical Epidemiology and Biostatistics, Faculty of Medicine, Ramathibodi Hospital, Mahidol University, Bangkok, Thailand; ^6^ Department of Orthopaedics, Faculty of Medicine, Srinakharinwirot University, Ongkharak, Nakhon Nayok, Thailand; ^7^ Centre for Public Health, School of Medicine, Dentistry, and Biomedical Sciences, Queen’s University, Belfast, Ireland; ^8^ School of Medicine and Public Health, University of Newcastle, Callaghan, NSW, Australia

**Keywords:** diabetes, anti-diabetic medications, GLP-1 agonists, SGLT-2 inhibitors, DPP-4 inhibitors, non-alcoholic fatty liver disease, NAFLD, network meta-analysis

## Abstract

**Objectives:**

This network meta-analysis aims to compare the efficacy and safety of new anti-diabetic medications for the treatment of non-alcoholic fatty liver disease (NAFLD).

**Materials and methods:**

PubMed and Scopus were searched from inception to 27^th^ March 2022 to identify all randomized controlled trials (RCTs) in NAFLD patients. Outcomes included reductions in intrahepatic steatosis (IHS) and liver enzyme levels. The efficacy and safety of DPP-4 inhibitors, GLP-1 agonists, SGLT-2 inhibitors, and other therapies were indirectly compared using a NMA approach. Unstandardized mean difference (USMD) with 95% confidence intervals (CI) were calculated.

**Results:**

2,252 patients from 31 RCTs were included. “Add-on” GLP-1 agonists with standard of care (SoC) treatment showed significantly reduced IHS compared to SoC alone [USMD (95%CI) -3.93% (-6.54%, -1.33%)]. Surface under the cumulative ranking curve (SUCRA) identified GLP-1 receptor agonists with the highest probability to reduce IHS (SUCRA 88.5%), followed by DPP-4 inhibitors (SUCRA 69.6%) and pioglitazone (SUCRA 62.2%). “Add-on” GLP-1 receptor agonists were also the most effective treatment for reducing liver enzyme levels; AST [USMD of -5.04 (-8.46, -1.62)], ALT [USMD of -9.84 (-16.84, -2.85)] and GGT [USMD of -15.53 (-22.09, -8.97)] compared to SoC alone. However, GLP-1 agonists were most likely to be associated with an adverse event compared to other interventions.

**Conclusion:**

GLP-1 agonists may represent the most promising anti-diabetic treatment to reduce hepatic steatosis and liver enzyme activity in T2DM and NAFLD patients. Nevertheless, longer-term studies are required to determine whether this delays progression of liver cirrhosis in patients with NAFLD and T2DM.

**Systematic review registration:**

https://www.crd.york.ac.uk/prospero/, identifier CRD42021259336.1.

## 1 Introduction

Non-alcoholic fatty liver disease (NAFLD) is a major cause of chronic liver disease worldwide. Its prevalence is estimated at ~33% of the global population (1, 2), and as high as 55% in those with type 2 diabetes mellitus (T2DM) (3). T2DM has been reported to accelerate the progression of NAFLD to more severe stages of non-alcoholic steatohepatitis (NASH), cirrhosis, and hepatocellular carcinoma (HCC), while the presence of NAFLD increases the risk of T2DM and makes achieving optimal glycemic control more difficult (4, 5).

NAFLD represents a broad spectrum of disease, ranging from liver steatosis to steatohepatitis, cirrhosis, and HCC (6). It is a multisystem disease that not only affects the liver, but carries extra-hepatic complications, including cardiovascular disease (CVD) leading to increased morbidity and premature mortality (7). The goal of NAFLD treatment is to reduce liver-related and cardiovascular morbidity/mortality. However, intermediate outcomes such as hepatic steatosis and hepatic fibrosis are strong predictors of disease progression. A significant percentage of individuals with hepatic steatosis will develop NASH which may progress to cirrhosis and HCC (8). Early diagnosis and treatment for diabetes associated NAFLD is recommended to delay disease progression and the onset of hepatic and extra-hepatic complications (9). Metabolic-associated fatty liver disease (MAFLD) is a recently proposed overarching terminology that addresses a spectrum of conditions associated with fatty liver disease and metabolic dysregulation. It more accurately reflects the underlying pathogenesis of the disease than NAFLD (10).

Despite the major health and economic burden, there is no FDA-approved pharmacological treatment. The clinical practice guidelines for NAFLD management recommend weight reduction and lifestyle modification as primary therapeutic options (6, 11, 12), but pharmacological treatments remain limited. For diabetic patients, pioglitazone, and glucagon-like peptide-1 receptor agonists (GLP-1 RAs) are recommended in T2DM with biopsy-proved NASH (6). However, concerns have been raised about the long-term safety of pioglitazone (13), limiting its widespread use.

The relationship between NAFLD, T2DM and inflammation are well-established (14, 15). New diabetes drugs have been reported to lower inflammatory markers (16–18). Thus, these medicines may have positive benefits in NAFLD by reducing inflammation. New generation anti-diabetic agents (such as dipeptidyl peptidase-4 inhibitors (DPP-4is), GLP-1 RAs, and sodium-glucose co-transporter-2 inhibitors (SGLT-2is)) have shown some potential benefits for NAFLD in previous randomized controlled trials (RCTs) (19–25). However, these RCTs had small sample sizes with inconsistent findings. Moreover, there has been no head-to-head comparison for pharmacologic management of NAFLD. Several systematic reviews and meta-analyses (SRMA) have evaluated the pairwise efficacy of medications for the treatment of NAFLD (26–28) but none have applied a network meta-analysis (NMA) approach.

NMA is a recent technique for comparing multiple treatments simultaneously in a single analysis by combining direct and indirect evidence (29). The method is very useful in clinical research, particularly for diseases with multiple treatment regimens and no head-to-head comparison. It also increases statistical power and allows for the ranking of therapies based on efficacy and safety (30). This NMA was undertaken to systematically compare and rank the efficacy and safety of the current regimens for the treatment of NAFLD in diabetic patients.

## 2 Materials and methods

This study was conducted in accordance with the Preferred Reporting Items for Systematic review and Meta-analysis (PRISMA) and the study protocol is registered on the PROSPERO website (CRD42021259336).

### 2.1 Data sources and search strategy

Initial searches were conducted in PubMed and Scopus from inception to 3^rd^ June 2021 with further updates until 27^th^ March 2022 without language restriction. Search terms were constructed according to the PICOS format for each database (**Table S1**) and the selection of included studies was conducted as follows. First, SRMAs were identified and RCTs included within these SRMAs were considered for inclusion in this study. Second, we searched for more recent individual RCTs published since the previous SRMA (26) through to 27^th^ March 2022. The bibliographies of relevant published trials and SRMAs were also considered to minimize the risk that relevant RCTs were overlooked.

### 2.2 Study selection

SRMAs and individual RCTs were identified by two independent reviewers (TK and TA); a third reviewer (VS or AT) was consulted in the event of any disagreement. SRMAs were selected if they met the eligibility criteria: (a) included only RCTs of adult NAFLD/NASH patients; (b) pooled treatment efficacy on hepatic outcomes and safety between any pair of new generation anti-diabetic agents and other treatments. Individual RCTs identified from selected SRMAs and additional RCTs published since the original search of the most recent SRMA were included if they met the following criteria; (a) studied adults aged ≥ 18 years with or without T2DM diagnosed with NAFLD or NASH; (b) compared any pair of DPP-4is, GLP-1 RAs, SGLT-2is and any other interventions (c) had at least one of the following outcomes: liver enzyme levels, intrahepatic steatosis or fibrosis, reported occurrence of cirrhosis or HCC, anthropometric data, metabolic profiles and any adverse events.

### 2.3 Data extraction

A data extraction form captured the following information: (a) article details (i.e., authors, year of publication, country, study design); (b) study characteristics (i.e., number and characteristics of participants); (c) baseline characteristics (i.e., age, number of T2DM/non-T2DM, sex, body weight (BW), body mass index (BMI), blood pressure, smoking); (d) treatments (i.e., type, route, dose and duration); (e) outcomes of interest (i.e., AST (SGOT), ALT (SGPT), intrahepatic steatosis). Data were independently extracted by two authors (TK and TA). Any discrepancy was resolved by discussion with a third reviewer (VS or AT).

### 2.4 Interventions, comparator, and outcomes of interest

Interventions of interest included DPP-4is, GLP-1 RAs, and SGLT-2is comparisons with standard of care (SoC) or other interventions (i.e., metformin, insulin, pioglitazone, omega-3, and lifestyle modification). Additional diabetic drugs that participants took for glycemic control at baseline within the placebo arm were considered as SoC in this analysis.

Outcomes of interest included change in intrahepatic steatosis evaluated by imaging (i.e., MRI, CT scan, ultrasound) or steatohepatitis determined by liver histology or liver enzyme levels (i.e., AST, ALT). Additional outcomes such as liver cirrhosis, HCC, anthropometric data (i.e., change of BW, BMI, waist circumference), and any medication side effects were also considered.

### 2.5 Risk of bias assessment

The revised Cochrane Risk-of-Bias tool version 2 (RoB2) was applied to assess the quality of RCTs (31). The RoB2 assesses five domains including the randomization process, deviations from the intended interventions, missing outcome data, measurement of the outcome, and selection of the result reported. Each study was considered as high or low risk of bias, or some concern according to the RoB2 scoring guide. Two reviewers (TK and PK) independently evaluated the study quality. If there was disagreement, a third adjudicator (TA) was consulted.

### 2.6 Statistical analysis

A pairwise MA, stratified by presence or absence of T2DM, was performed if at least 3 studies compared outcomes between the same treatment pair. Treatment effects (i.e., unstandardized mean difference (USMD) for continuous outcomes and risk ratio (RR) for dichotomous outcomes) were estimated and pooled across studies using a random-effect model if heterogeneity was present, otherwise a fixed-effect model was applied. Heterogeneity was checked using Q-test and I^2^ statistics and was considered present if the Q test was significant and/or I^2^ > 25% (32). A meta-regression was applied to explore sources of heterogeneity.

A two-stage NMA framework approach was applied. First, a regression analysis estimated relative treatment effects (i.e., lnRR, risk difference (RD), USMD) along with measures of variance-covariance. Second, these were pooled across studies using a multivariate MA model with consistency. The inconsistency assumption was assessed by global approaches using design-by-treatment interaction models. A loop-specific approach was applied to estimate an inconsistency factor (IF) if the inconsistency assumption did not hold. Treatments were ranked using a rankogram and surface under the cumulative ranking curve (SUCRA). Publication bias was assessed using comparison-adjusted Funnel plots and Egger’s tests. Sensitivity analyses explored the effect of studies with high risk of bias.

STATA version 17 (StataCorp, Texas, USA) was used for statistical analyses. A significance threshold p-value < 0.05 was considered for all analyses, except the heterogeneity and Egger's tests, where a p-value < 0.10 was considered more appropriate.

## 3 Results

**Figure 1:**
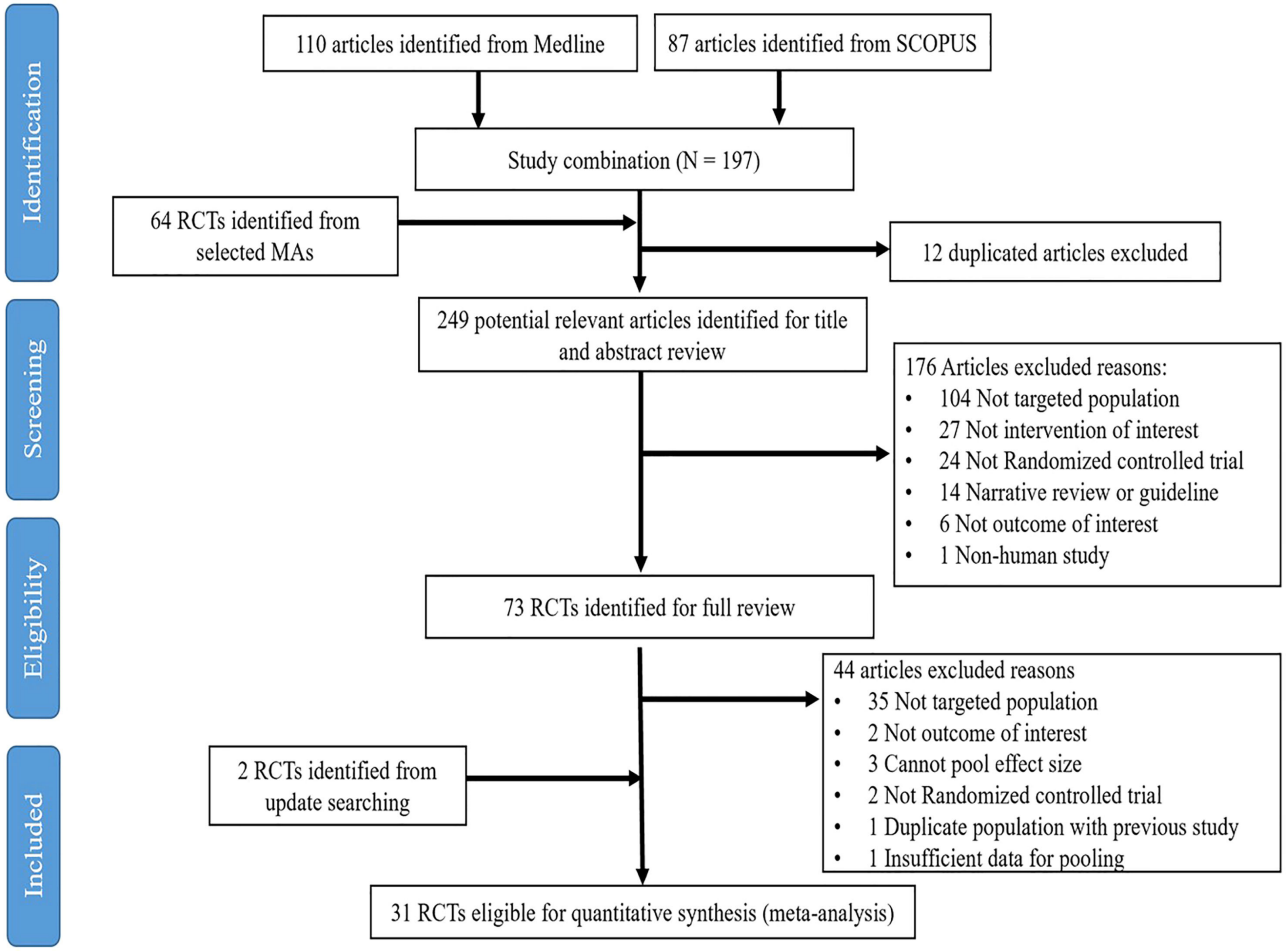
Prisma flow diagram.

Of the 64 RCTs identified from the SRMAs and 197 individual articles from the updated search, only 31 (n=2,252) met the inclusion criteria and were retained in the final analysis (see **Figure 1**). Of these, 22 (n=1,521), 4 (n=202), and 5 RCTs investigated T2DM, non-T2DM, and a mix of T2DM/non-T2DM patients (n=529), respectively. For non-T2DM patients, there was insufficient data to make a valid comparison [GLP-1 RAs and diet+exercise (2 studies, n=54), DPP-4is and placebo (1 study, n=58), and SGLT-2is and placebo (1 study, n=90)]. Of the RCTs composed of mixed T2DM/non-T2DM participants, 3 studies had >50% of participants with T2DM and it was therefore decided to combine them with the 22 T2DM studies, providing 25 RCTs in the final analysis. Of these, 4, 10, and 11 RCTs evaluated DPP-4is (n=184), SGLT-2is (n=635) and GLP-1 RAs (n=1,139), respectively. Baseline characteristics are presented in **Table 1**. Mean age varied from 29.5 to 65.5 years, and percentage of male participants ranged between 15.0% and 91.7%. Treatment duration ranged between 12 and 72 weeks with a median value of 24 weeks. The proportion of patients with NASH was only reported in two studies at baseline (n=332).

**Table 1:** RCT study characteristics.

Study	Country	Study Population		Total Population (n)	Mean age (year)	Male (%)	BMI (kg/m^2^)	AST/ALT/GGT (IU/L)	Intrahepatic steatosis	Intervention	Comparator	Follow up (weeks)	Primary outcome
		NASH/NAFLD	DM/non-DM										
DPP-4 inhibitor													
Cui 2016 (33)	USA	NAFLD	T2DM	50	53.9	42	31.8	28.5/41.5/33.0	17.5% (MRI-PDFF)	Sitagliptin	Standard treatment for T2DM	24	MRI-PDFF
Hussain 2016 (34)	Pakistan	NAFLD	Non-DM	58	29.5	65.5	30.2	67.8/68.1/19.7	–	Vildagliptin	Placebo	12	Biochemical, metabolic and fatty changes
Deng 2017 (35)	China	NAFLD	T2DM	72	63.9	75.0	23.3	33.6/35.1/-	–	Sitagliptin	Diet and exercise	52	Biochemical profiles
Joy 2017 (36)	UK	NASH (biopsy-proven)	T2DM	12	55.7	41.5	36.7	41.5/59.0/132.0	–	Sitagliptin	Standard treatment for T2DM	24	Liver fibrosis on histology
Alam 2018 (37)	Bangladesh	NASH (biopsy-proven)	Both DM and non-DM	40	38.6	30	26.5	42.2/62.5/53.6	–	Sitagliptin	Placebo	52	Change in steatosis, inflammation, fibrosis and NAS in liver biopsy
Komorizono 2021 (38)	Japan	NAFLD	T2DM	50	52.6	38.8	28.8	34.3/45.9/43.4	41 HU (CT scan)	Linagliptin	Metformin	52	Hepatic steatosis evaluated by CT-HU
SGLT-2 inhibitor													
Ito 2017 (39)	Japan	NAFLD	T2DM	66	58.2	48.5	30.3	41.6/55.2/67.3	0.79 (L/S ratio by CT)	Ipragliflozin	Pioglitazone	24	L/S ratio by CT scan
Shibuya 2018 (40)	Japan	NAFLD	T2DM	32	55.5	56.3	27.6	-/24.8/-	0.95 (L/S ratio by CT)	Luseogliflozin	Metformin	24	L/S ratio by CT scan
Eriksson 2018 (41)	Sweden	NAFLD	T2DM	84	65.5	70.2	31.2	30.4/36.6/45.6	17.9% (MRI-PDFF)	Dapagliflozin	Omega-3, placebo	12	MRI-PDFF
Kuchay 2018 (42)	India	NAFLD	T2DM	50	49.9	59.5	29.7	45.0/64.8/64.9	16.3% (MRI-PDFF)	Empagliflozin	Standard treatment for T2DM	20	MRI-PDFF
Shimizu 2019 (21)	Japan	NAFLD	T2DM	63	56.6	59.7	27.9	27.2/35.9/43.0	–	Dapagliflozin	Standard treatment for T2DM	24	Fibroscan and CAP measurement
Han 2020 (43)	Korea	NAFLD	T2DM	45	53.9	62.2	30.3	27.7/32.3/37.6	–	Ipragliflozin	Pioglitazone+ metformin	24	Total visceral fat, CAP, fatty liver index
Taheri 2020 (22)	Iran	NAFLD	Non-DM	90	44	55.5	30.3	25.3/36.1/-	–	Empagliflozin	Placebo	24	Hepatic steatosis and fibrosis (elastrography)
Kinoshita 2020 (44)	Japan	NAFLD	T2DM	98	59	45.9	28.8	35.0/47.2/54.9	0.74 (L/S ratio by CT)	Dapagliflozin	Pioglitazone, glimepiride	28	L/S ratio by CT scan
Cho 2021 (45)	Japan	NAFLD	T2DM	53	63.5	15	–	23.3/22.2/25.6	–	Dapagliflozin	Pioglitazone	24	Liver fat index
Chehrehgosha 2021 (46)	Iran	NAFLD	T2DM	106	51.6	43.4	30.2	22.8/30.7/-	–	Empagliflozin	Pioglitazone, Standard treatment for T2DM	24	Liver fat content and liver stiffness using fibroscan
Yoneda 2021 (47)	Japan	NAFLD	T2DM	38	58.6	52.5	30.0	58.8/81.8/78.7	17.6% (MRI-PDFF)	Tofogliflozin	Pioglitazone	24	MRI-PDFF
GLP-1 receptor agonist													
Fan 2013 (48)	China	NAFLD	T2DM	117	52.4	56.4	27.0	35.0/65.8/65.8	–	Exenatide	Metformin	12	Biochemical and metabolic profile
Shao 2014 (49)	China	NAFLD with elevated liver enzyme	T2DM	60	43.0	51.2	31.0	123.5/166.7/135.7	–	Exenatide	Insulin	12	Metabolic change, hepatic injury biomarkers, fatty liver
Armstrong 2016 (19)	UK	NASH (biopsy-proven)	Both DM and non-DM	52	51.0	57.5	36.0	71.5/51.0/103.0	–	Liraglutide	Placebo	48	Resolution of NASH
Feng 2017 (50)	China	NAFLD	T2DM	87	47.0	68.9	27.6	31.3/48.6/-	–	Liraglutide	Metformin, gliclazide	24	Fatty liver evaluated by ultrasound
Khoo 2017 (51)	Singapore	NAFLD with elevated liver enzyme	Non-DM	24	41.4	91.7	33.1	52.5/92.5/-	30.2% (liver fat fraction-MRI)	Liraglutide	Diet and exercise	26	Liver fat fraction (MRI)
Tian 2018 (52)	China	NAFLD	T2DM	127	57.3	58.2	27.8	35.0/65.8/-	–	Liraglutide	Metformin	12	Biochemical and metabolic profile
Yan 2019 (20)	China	NAFLD	T2DM	75	44.8	69.3	29.8	33.0/43.0/-	15.3% (MRI-PDFF)	Liraglutide	Insulin glargine, sitagliptin	26	MRI-PDFF
Khoo 2019 (53)	Singapore	NAFLD with elevated liver enzymes	Non-DM	30	40.7	90	33.2	48.5/87.5/-	31.1% (liver fat fraction-MRI)	Liraglutide	Diet and exercise	26	Liver fat fraction (MRI)
Zhang 2020 (54)	China	NAFLD	T2DM	60	50.9	46.7	27.4	33.1/33.3/24.7	24.0% (H-MRS)	Liraglutide	Pioglitazone	24	H-MRS
Liu 2020 (55)	China	NAFLD	T2DM	71	49.1	53.5	28.2	28.2/37.7/59.3	38.8% (H-MRS)	Exenatide	Insulin	24	H-MRS
Guo 2020 (23)	China	NAFLD	T2DM	91	52.6	55.9	28.7	28.6/31.8/-	25.7% (H-MRS)	Liraglutide	Metformin, insulin	26	H-MRS
Kuchay 2020 (56)	India	NAFLD	T2DM	64	47.4	70.5	29.8	35.0/65.8/66.4	17.5% (MRI-PDFF)	Dulaglutide	Standard treatment for T2DM	24	MRI-PDFF
Newsome 2021 (57)	Multicenter	NASH (biopsy-proven)	Both DM and non-DM	320	55.0	39.3	33.8	43.3/54.3/62.8	–	Semaglutide	Placebo	72	Resolution of NASH
Flint 2021 (58)	Germany	NAFLD	Both DM and non-DM	67	60.0	70.1	–	37.0/30.0/-	17.7% (MRI-PDFF)	Semaglutide	Placebo	72	Liver stiffness by MRE

L/S ratio, liver-to-spleen attenuation ratio; CT-HU, computed tomography imaging-estimated Hounsfield units; CAP, controlled attenuation parameter; MRI-PDFF, Magnetic resonance imaging-proton density fat fraction; H-MRS, Proton magnetic resonance spectroscopy; MRE, Magnetic resonance elastrography.

### 3.1 Effect of intervention on intrahepatic steatosis

**Figure 2:**
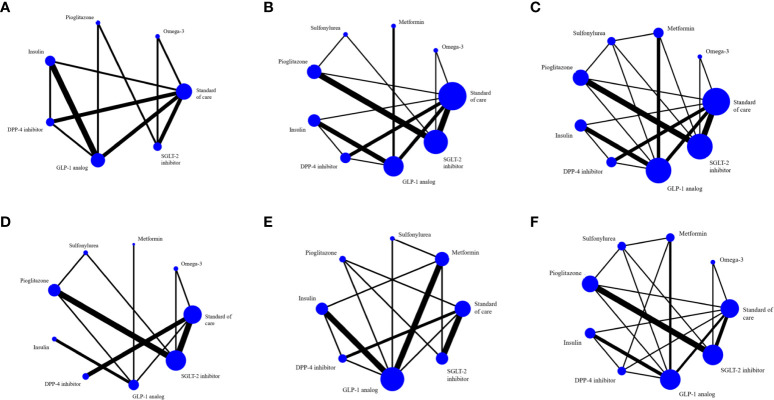
The evidence network includes studies on **(A)** MRI-based intrahepatic fat, **(B)** SGOT, **(C)** SGPT, **(D)** GGT, **(E)** BMI and **(F)** any adverse events. The entire sample size of the related intervention is shown by the size of the nodes (blue circles). Each line depicts a direct comparison of the two interventions, with the thickness of the line corresponding to the number of trials that evaluated the comparison.

There were 3, 4 and 12 studies that used ultrasound, CT, and MRI respectively to measure intrahepatic fat but only the 12 MRI studies (MRI-PDFF and ^1^H-MRS) provided sufficient data for analysis. Three studies (20, 23, 55) (n=180) compared GLP-1 RAs with insulin on intrahepatic steatosis; a pairwise MA provided a pooled USMD (95% CI) of -1.71% [(-3.68%, 0.26%); P =0.09; I^2 ^= 0%] suggesting GLP-1 RAs reduced intrahepatic steatosis by 1.71% compared to insulin, (**Figure S1A**). A NMA compared 6 treatment effects on intrahepatic steatosis relative to SoC (i.e., SGLT-2is, GLP-1 RAs, DPP-4is, insulin, pioglitazone, omega-3), see **Figure 2A**. Although all treatments improved intrahepatic steatosis relative to SoC, only GLP-1 RAs was significant with a pooled USMD (95% CI) of -3.93% (-6.54%, -1.33%). GLP-1 RAs also tended to reduce intrahepatic steatosis compared with pioglitazone, but this was not significant [USMD (95%CI) of 1.42% (-1.76%, 4.59%)], see **Table 2**. The SUCRA ranking identified “add-on” GLP-1 RAs to SoC as the best option for reducing intrahepatic steatosis in T2DM patients (SUCRA 88.5%) followed by DPP-4is (SUCRA 69.6%) and pioglitazone (SUCRA 62.2%), see **Table S2** and **Figure S2A**.

**Table 2:** Relative treatment effect comparisons (95%CI) for in intrahepatic steatosis evaluated by MRI in diabetes patients.

***SoC***	-1.34 (-4.52,1.84)	-3.93* (-6.54, -1.33)	-2.95 (-6.99,1.08)	-2.25 (-5.37,0.87)	-2.52 (-6.01,0.98)	3.82 (-2.89,10.52)
***SGLT-2i***		-2.59 (-6.22, 1.03)	-1.61 (-6.47,3.25)	-0.91 (-5.07,3.25)	-1.18 (-4.73,2.38)	5.16 (-1.73,12.05)
***GLP-1 RA***			0.98 (-2.54,4.50)	1.68 (-1.01,4.38)	1.42 (-1.76,4.59)	7.75* (0.63,14.87)
***DPP-4i***				0.70 (-3.05,4.45)	0.43 (-4.32,5.19)	6.77 (-1.03,14.57)
***Insulin***					-0.27 (-4.27,3.74)	6.07 (-1.28,13.42)
***Pioglitazone***						6.33 (-0.99,13.65)
***Omega-3***						

WMD, weight mean difference; SoC, standard of care; SGLT2i, SGLT-2 inhibitor; GLP-1 RA, GLP-1 receptor agonist; DPP-4i, DPP-4 inhibitor; *statistical significance.

### 3.2 Effect of intervention on liver enzymes

Five studies (21, 41–43, 46) (n=255) directly compared SGLT-2is with SoC alone. A pairwise MA identified that “add-on” SGLT-2is significantly reduced ALT compared to SoC, with a pooled USMD of -4.81 (-7.82, -1.81) U/L; P < 0.01; I^2 ^= 0%. However, no significant changes in AST and GGT were observed, with pooled USMDs of -1.93 (-4.27, 0.41) U/L; P=0.1; I^2 ^= 0% and 0.42 (-4.51, 5.35) U/L; P =0.87; I^2 ^= 24.95%, respectively, see **Figures S1B, C, D**. Five studies (39, 44–47) comparing SGLT-2is and pioglitazone (N=291) showed no significant effect on any liver enzyme activity. Studies (20, 23, 49, 55) of GLP-1 RAs (N=240) and insulin showed significantly reduced AST by -5.76 [(-8.89, -2.62) U/L; P < 0.01; I^2 ^= 59.46%] and reduced GGT by -9.27 [(-13.05, -5.49) U/L; p < 0.01; I^2 ^= 0%] but no significant effect on ALT by -7.33 [(-19.76, 5.11) U/L; P < 0.01; I^2^ = 89.00%], see **Figures S1B, C, D**.

A NMA framework consisted of 22 RCTs comparing eight treatments on AST (omega-3, metformin, sulfonylurea, pioglitazone, insulin, DPP-4is, SGLT-2is, GLP-1 RAs) with SoC, see **Figure 2B** and **Table 3**. The consistency assumption did not hold (X^2^ = 54.3, P <0.001), and the loop-specific approach identified the sulfonylurea-pioglitazone-GLP-1 RAs loop as having a high IF of 17.01. After exclusion of two studies (50, 54), network consistency was improved (X^2^ = 13.9, P=0.09). The final network of 20 studies (**Figure 2B**) showed that all treatments tended to reduce AST activity compared to SoC with the exception of sulfonylurea and omega-3, but only GLP-1 RAs exceeded the significance threshold with a pooled USMD (95%CI) of -5.04 (-8.46,-1.62) U/L. Compared with pioglitazone, GLP-1 RAs tended to lower AST activity, although not significantly [USMD (95%CI) of -3.2(-8.05, 1.65)] U/L, see **Table 3**. The SUCRA identified the top ranked intervention as GLP-1 RAs (97.2%), followed by SGLT-2is (74%) and DPP-4is (68.8%) (**Table S2**).

**Table 3:** Relative treatment effect comparisons (95%CI) for AST (upper triangle) and ALT (lower triangle).

***SoC***	-2.23 (-4.99,0.54)	-5.04* (-8.46,-1.62)	-2.15 (-6.89,2.58)	0.94 (-2.96,4.84)	-1.84 (-5.28,1.60)	2.77 (-4.45,9.99)	0.89 (-3.71,5.50)	6.62 (-0.55,13.79)
-1.69 (-8.21,4.82)	***SGLT-2 inhibitor***	-2.81 (-7.26,1.63)	0.07 (-5.43,5.57)	3.17 (-1.63,7.97)	0.38 (-2.32,3.09)	5.00 (-1.83,11.82)	3.12 (-2.29,8.53)	8.85* (1.75,15.95)
-9.84* (-16.84,-2.85)	-8.15* (-16.05,-0.25)	***GLP-1 agonist***	2.89 (-1.88,7.66)	5.98* (2.48,9.48)	3.20 (-1.65,8.05)	7.81 (-0.28,15.90)	5.93* (2.85,9.02)	11.66* (3.72,19.61)
1.06 (-9.39,11.51)	2.75 (-9.13,14.63)	10.90 (-0.13,21.93)	***DPP-4 inhibitor***	3.10 (-2.14,8.34)	0.31 (-5.54,6.16)	4.92 (-3.76,13.61)	3.05 (-2.63,8.73)	8.78* (0.18,17.37)
-0.13 (-9.55,9.29)	1.56 (-8.88,12.00)	9.71* (1.73,17.69)	-1.19 (-13.23,10.85)	***Insulin***	-2.79 (-7.99,2.42)	1.83 (-6.43,10.08)	-0.05 (-4.71,4.62)	5.68 (-2.49,13.84)
-4.96 (-12.94,3.01)	-3.27 (-9.76,3.22)	4.88 (-3.56,13.33)	-6.02 (-18.58,6.55)	-4.83 (-15.82,6.16)	***Pioglitazone***	4.61 (-2.02,11.25)	2.74 (-3.01,8.48)	8.46* (0.96,15.97)
3.23 (-9.25,15.71)	4.92 (-7.11,16.95)	13.08* (1.21,24.94)	2.18 (-13.35,17.71)	3.36 (-10.64,17.37)	8.19 (-4.04,20.43)	***Sulfonylurea***	-1.88 (-10.53,6.78)	3.85 (-5.95,13.66)
-0.29 (-9.80,9.22)	1.40 (-7.95,10.75)	9.55* (1.63,17.47)	-1.35 (-14.43,11.74)	-0.16 (-11.17,10.85)	4.67 (-5.53,14.87)	-3.53 (-16.05,9.00)	***Metformin***	5.73 (-2.80,14.25)
10.41 (-4.85,25.66)	12.10 (-3.22,27.42)	20.25* (3.89,36.62)	9.35 (-9.00,27.71)	10.54 (-7.09,28.17)	15.37 (-0.93,31.67)	7.18 (-11.87,26.23)	10.70 (-6.66,28.06)	***Omega-3***

Comparison should be read from right to left. In the upper triangle; mean difference < 0 favors drug in the column. In the lower triangle, mean difference > 0 favors drug in the column, *statistical significance.

For ALT activity, 22 studies that considered 8 interventions (omega-3, metformin, sulfonylurea, pioglitazone, insulin, DPP-4is, SGLT-2is, GLP-1 RAs) were pooled without evidence of inconsistency (X^2^ = 10.8, P 0.55), see **Figure 2C**. All comparators, except sulfonylurea and omega-3, added-on to SoC tended to lower ALT compared with SoC alone, but only GLP-1 RAs reached significance with a pooled USMD of -9.84 (-16.84, -2.85) U/L. Compared with pioglitazone, GLP-1 RAs tended to reduce ALT, but not significantly [USMD (95%CI) of -4.88(-13.33, 3.56)], see **Table 3**. Based on SUCRA, the three top ranked medications were GLP-1 RAs (97.2%), pioglitazone (77.0%) and SGLT-2is (57.3%) (**Table S2**).

Sixteen studies with 8 treatments (omega-3, metformin, sulfonylurea, pioglitazone, insulin, DPP-4is, SGLT-2is, GLP-1 RAs) were considered for GGT activity (**Figure 2D**) without evidence of inconsistency (X^2 ^=^ ^4.03, P 0.26). GLP-1 RAs and metformin significantly reduced GGT with pooled USMDs (95%CI) of -15.53 (-22.09, -8.97) and -9.61 (-18.28, -0.94) U/L, respectively (**Table S3**). SUCRA identified the top ranked treatment as GLP-1 RAs (99.6%) followed by SGLT-2is (64.5%) and metformin (61.4%) (**Table S2**, **Figure S2D**).

### 3.3 Effect of intervention on BMI

Pooling four studies (23, 48, 50, 52) (n=363) indicated that GLP-1 RAs significantly lowered BMI compared to metformin with an USMD of -0.96 [(-1.36, -0.56) kg/m^2^; P < 0.01; I^2^ = 13.36%)]. A NMA approach compared the effect of seven treatments on BMI (i.e., SGLT-2is, GLP-1 RAs, DPP-4is, insulin, pioglitazone, metformin, sulfonylurea) relative to SoC, see **Figure 2E**. All treatments tended to reduce BMI compared to SoC, but none were significant. However, GLP-1 RAs significantly reduced BMI compared with pioglitazone [USMD (95%CI) of -2.33 (-4.15, -0.31) kg/m^2^]. The SUCRA identified that “add-on” GLP-1 RAs to SoC was best in reducing BMI in diabetic patients (95.3%) followed by SGLT-2is (64.5%) and metformin (61.4%), see **Table S2** and **Figure S2E**.

### 3.4 Additional outcomes

#### 3.4.1 Resolution of NASH

Only two RCTs reported NASH remission based on liver biopsy. Armstrong et al. found that liraglutide improved some features of liver histology in a small study (n=52) (19). Recently, a phase two RCT that included 320 NASH patients evaluated daily dosages of semaglutide with placebo. Resolution of steatohepatitis was found in 40%, 36%, 59% and 17% in 0.1 mg, 0.2 mg, 0.4 mg and placebo groups, respectively. However, the percentage of people with improved fibrosis did not significantly differ across groups (57).

### 3.5 Adverse effects from medications

Seventeen studies reported mild and non-life-threatening adverse effects in T2DM such as gastrointestinal disorders (nausea, vomiting, diarrhea), hypoglycemia for GLP-1 RAs; urinary tract infection, genital tract infection, euglycemic ketoacidosis, polyuria for SGLT-2is; pancreatitis, dyspepsia, constipation for DPP-4is. A NMA considered eight interventions (omega-3, metformin, sulfonylurea, pioglitazone, insulin, DPP-4is, SGLT-2is, GLP-1 RAs; **Figure 2F**) indicating that sulfonylurea, pioglitazone, and SGLT-2is had lower adverse events than SoC but only sulfonylurea was significant [RD (95% CI) of -0.22 (-0.39, -0.05)]; by contrast, other treatments tended to have more adverse events but only GLP-1 RAs was significant [RD (95%CI) of 0.17 (0.06, 0.28)]. In addition, sulfonylurea had significantly less adverse events than other active drugs with RDs (95% CI) of -0.17 (-0.33, -0.00), -0.39 (-0.55, -0.22), -0.23 (-0.43, -0.02), -0.31 (-0.52, -0.11), and -0.20 (-0.37, -0.03) for SGLT-2is, GLP-1 RAs, DPP-4is, insulin, and pioglitazone, respectively (**Table S5**). According to the SUCRA, GLP-1 RAs had the most adverse events, whereas sulfonylureas had the least.

### 3.6 Risk of bias assessment

The RoB assessment is summarized in **Figure S3**. Seven studies were considered at high risk of bias, 18 studies raised some concerns and only 6 studies were considered low risk of bias. The two domains with the poorest ratings were the randomization method and deviation from the interventions intended.

### 3.7 Publication bias

No evidence of asymmetry in comparison-adjusted funnel plots was identified in any of the six networks, see **Figure S4**.

A sensitivity analysis to exclude studies with a high risk of bias could not be performed because of the variety of interventions and comparators that were employed in the included trials.

## 4 Discussion

We performed a NMA including 31 RCTs to assess the efficacy of eight diabetic treatments (i.e., SGLT-2is, GLP-1 RAs, DPP-4is, insulin, pioglitazone, sulfonylurea, metformin, omega-3) relative to SoC. Our study included more RCTs and more participants compared to the most recent pairwise MA (27). Moreover, we only considered studies that included diabetic participants and compared antidiabetic agents. We demonstrate that GLP-1 RAs were most likely to reduce intrahepatic steatosis, liver enzyme levels, and BMI compared to other interventions for diabetic NAFLD patients.

In general, liver biopsy is considered the gold standard for confirming NASH diagnosis and assessing the severity of liver fibrosis. However, the invasiveness and feasibility of performing liver biopsy limits the number of clinical trials that consider this clinical outcome. Several biomarkers including imaging, composite scores and liver profiles have therefore been considered as proxy outcome measures in clinical trials (6).

Multiparametric MRI is now commonly considered in clinical settings and academic research. Although several techniques can quantify the amount of liver fat in an MRI scan,^1^H-MRS and PDFF are the methods that are most frequently employed. PDFF measurement is the current gold standard for MRI assessment of hepatic fat content (59). When compared to steatosis grading on a histological basis, PDFF accurately reflects the triglyceride concentration in liver tissue with strong intra- and inter-observer agreement (60). PDFF also significantly correlated with histologic steatosis grading with an area under the curve (AUROC) of 0.90-0.94 in a prospective validation study (61). The 12 MRI trials (MRI-PDFF and ^1^H-MRS) included in this NMA showed that GLP-1 RAs significantly decreased IHS when compared to SoC alone, and they had similar, but non-significant effects, when compared to pioglitazone. The results were comparable with the prior SRMA, which found that GLP-1 RAs significantly reduced IHS on MRI-based techniques of -3.92% (95%CI: -6.27%, -1.56%) (62). Several potential mechanisms by which GLP-1 RAs reduce IHS have been proposed. The benefits might be directly associated with an effect on hepatocyte and hepatic metabolism i.e. enhanced ß-cell function, promoting hepatic insulin sensitivity, alteration of genes related to fatty acid oxidation and *de novo* lipogenesis in the liver (63–67) and indirect association with body weight reduction (68).

Elevated plasma AST levels have shown good correlation with increased risk of developing advanced stages of liver disease (69). Elevated serum ALT has also been reported in association with increased hepatic fat content (70). AST/ALT ratio is also a reasonable predictor of advanced fibrosis (71). Elevated GGT is associated with an increased risk of several diseases, including cardiovascular disease, diabetes, metabolic syndrome, NAFLD and all-cause mortality (72). In accordance with previously SRMA (62, 73), this study identified that “add-on” GLP-1 RAs significantly reduces IHS and liver enzyme activity compared to SoC alone. We also demonstrated that GLP-1 RAs showed better hepatic outcomes compared to SGLT-2is (significantly reduced liver enzymes and a trend towards improved liver steatosis on imaging) and DPP-4is (a trend towards improved liver steatosis and liver enzyme activity). When compared with pioglitazone, GLP-1 RAs tends to lower ALT activity in both direct and indirect comparisons although it fails to reach the significance threshold. Pioglitazone has been shown to improve liver enzyme activity in NASH patients (26). Nevertheless, only four studies that investigated patients with NASH at baseline were included in this analysis. SGLT-2is and DPP-4is showed a trend for improved liver enzyme profiles for both direct and indirect comparisons compared to SoC alone, although this was not significant. However, there is no association between the reduction of liver enzyme and long-term health outcomes.

The association between weight reduction and liver outcomes was not observed in our study. Nevertheless, our NMA suggests that GLP-1 RAs and SGLT-2is provide the highest probability of reducing BMI, although not significantly compared to SoC. There could be several explanations for this finding. Firstly, there was only one direct comparison between GLP-1 agonist (dulaglutide 0.75-1.5 mg weekly) with SoC (n/N=52/1) in this NMA, providing limited power to evaluate any differences. Second, the average treatment duration was only 20.4 months (range 12-26 weeks), in which participants in three (n=304) of the nine included studies (n=752) had only been on GLP-1 RAs for only 12 weeks. Third, the mean baseline BMI of included participants was relatively low (28.6, range 27.0-31.0 kg/m^2^) compared to other studies of GLP-1 RAs (BMI 33.8-36.0 kg/m^2^) (19, 51, 53, 57). Fourth, all participants had NAFLD at baseline and could have been advised to reduce weight for their condition. The benefits of diet and exercise for weight loss in the SoC group are not taken into account in this analysis.

With the increasing prevalence of NAFLD and the high burden associated with hepatic-related and cardiovascular mortality, pharmaceutical interventions that would provide additional benefits beyond glycemic control to delay hepatic progression might reduce premature mortality in patients with diabetes associated NAFLD (25). SGLT-2is and GLP-1 RAs have shown promise for improved cardiovascular outcomes and are the recommended first line medications for T2DM patients with established or at high risk of CVD (74). For T2DM with NAFLD, GLP-1 RAs could be considered a primary treatment option given the hepatic benefit identified in the present study by reducing AST, ALT, and GGT of about 5, 9, 15 U/L, respectively.

A novel dual glucose dependent insulinotropic polypeptide (GIP) and GLP-1 agonist (Tirzepatide) is a new anti-diabetic agent that was recently approved for glycemic control in T2DM patients (75). It has been investigated in a sub-study of the SURPASS (A study of Tirzepatide in participants with type 2 diabetes not controlled with diet and exercise alone) phase 3 clinical trial program. Tirzepatide 10 mg and 15 mg resulted in 8.21% and 7.78% decreased liver fat content, as measured by MRI-PDFF, over a 52-week-period, respectively (76). In addition, Tirzepatide also significantly reduced ALT and AST activity from baseline at 26 weeks in a phase 2 study (n=316) (77). Thus, dual GIP and GLP-1 agonist offer potential benefit for the treatment of NAFLD/NASH. However, given the complex etiology of NAFLD, the use of this agent requires a thorough clinical patient-centered approach and more well-designed studies to avoid potential confounding bias (68).

There are several strengths to our study. First, to the best of our knowledge, this is the first NMA to provide a quantitative assessment of the efficacy of novel anti-diabetic agents (SGLT-2is, DPP-4is and GLP-1 RAs). Second, this study included a broad range of participants and interventions (diabetic and non-diabetic patients, NAFLD and NASH, and pharmacological and non-pharmacological therapies). Third, this study included only RCTs which represent the highest level of evidence. Fourth, this study had the largest number of RCTs and patients (31 studies, 2,252 patients) compared to previous NAFLD SRMAs (26 studies, 946 patients) (26). Moreover, this study clearly defined the effect size for each comparator and demonstrated the efficacy of novel antidiabetic treatment options for comparison with other NAFLD interventions.

Our study also had several limitations. First, this study used aggregated study-level data as opposed to individual participant data from different populations. Consequently, we could not evaluate baseline factors that may have influenced treatment effects. Second, studies included were subject to heterogeneity with respect to treatment duration, primary end points, and assessment of treatment efficacy, with only a limited number of studies using liver imaging modalities and liver biopsy as the “gold standard” for NASH grading. Third, treatment-effects may have been affected by the concomitant use of other antidiabetic medication, especially pioglitazone, which has been shown to improve both liver histology and liver enzyme activity (78). Fourth, there were insufficient data to investigate long-term outcomes such as fibrosis, cirrhosis, and death. Fifth, treatment effects were assessed as drug classes, there was no distinction made between individual medicines in the drug classes (e.g., exenatide vs. liraglutide vs. semaglutide) or different doses. Sixth, several studies included (7 of the 31) were at high risk of bias. As such, larger RCTs with longer periods of follow-up would help address these shortcomings.

## 5 Conclusions

GLP-1 receptor agonists may represent the most promising treatment option for improving hepatic steatosis and liver enzyme levels (AST, ALT, GGT) in patients with NAFLD. However, the supporting evidence is limited by sample size and variability in outcome measures. High-quality RCTs with larger sample sizes and longer follow-up times are warranted to confirm these findings and maximize the therapeutic benefit from these treatment options.

## Data availability statement

The original contributions presented in the study are included in the article/**Supplementary Material**. Further inquiries can be directed to the corresponding authors.

## Author contributions

TK designed the study, collected the data, contributed to the statistical analysis, risk of bias assessment, writing the manuscript and served as the primary author. TA contributed to the study design, data collection, statistical analysis and provided critical feedback to develop the manuscript. PK contributed to risk of bias assessment. GM and JA helped develop the manuscript and provided critical feedback. AT and VS contributed to the study design, statistical analysis and provided critical feedback in writing the manuscript. All authors contributed to the article and approved the submitted version.
