# Supplementary material for: New anti-diabetic agents for the treatment of non-alcoholic fatty liver disease: a systematic review and network meta-analysis of randomized controlled trials
Source: Front Endocrinol (Lausanne). 2023 Jun 27;14:1182037. doi: 10.3389/fendo.2023.1182037 (PMC10335801; doi:10.3389/fendo.2023.1182037)
Supplement: Supplementary file 1 [file DataSheet_1.docx]

Supplementary Material

**New anti-diabetic agents for the treatment of non-alcoholic fatty liver disease: a systematic review and network meta-analysis of randomized controlled trials.**

**Tanawan Kongmalai, Varalak Srinonprasert^*^, Thunyarat Anothaisintawee, Pinkawas Kongmalai, Gareth McKay, John Attia, Ammarin Thakkinstian^*^**

*** Correspondence:** Varalak Srinonprasert (E-mail: [Varalak.sri@mahidol.edu](mailto:Varalak.sri@mahidol.edu), [Varalaksi@gmail.com](mailto:Varalaksi@gmail.com)), Ammarin Thakkinstian (E-mail**:** [Ammarin.tha@mahidol.edu](mailto:Ammarin.tha@mahidol.edu)).

**List of supplementary**

**Supplementary Table S1:** Electronic search strategies

**Supplementary Table S2.** SUCRA (%) for the efficacy of each treatment on intrahepatic steatosis, liver enzymes, body mass index (BMI) and any adverse event in T2DM patients with NAFLD.

**Supplementary Table S3**. Relative treatment effects by mean difference on GGT: A network meta-analysis.

**Supplementary Table S4.** Relative treatment effects by mean difference on body mass index (BMI): A network meta-analysis.

**Supplementary Table S5**. Relative treatment effects by risk difference on any adverse event: A network meta-analysis.

**Supplementary Figure S1:** Forrest plots of pairwise meta-analysis

**Supplementary Figure S2:** SUCRA graph of each outcome

**Supplementary Figure S3:** Risk of bias assessment for studies included.

**Supplementary Figure S4:** Comparison-adjusted funnel plots

**Supplementary Table S1:** Electronic search strategies

- **Search strategy in Medline databased (search date 3^rd^ June 2021)**

| **Domains** | **Search number** | **Search term** | **Results (studies)** |
| --- | --- | --- | --- |
| Population | #1 | "nonalcoholic fatty liver" | 12,035 |
|  | #2 | "non-alcoholic fatty liver" | 20,545 |
|  | #3 | (#1) OR (#2) | 26,070 |
|  | #4 | "non-alcoholic" AND "fatty liver" | 21,338 |
|  | #5 | "non-alcoholic fatty liver disease"[MeSH Terms] | 14,696 |
|  | #6 | (#3) OR (#5) | 26,070 |
|  | #7 | "NAFLD" | 24,903 |
|  | #8 | "nonalcoholic steatohepatitis" | 6,200 |
|  | #9 | “non-alcoholic steatohepatitis” | 4,742 |
|  | #10 | (#8) OR (#9) | 10,635 |
|  | #11 | “NASH” | 20,034 |
|  | #12 | (#10) OR (#11) | 23,610 |
|  | #13 | (#1) OR (#2) OR (#4) OR (#5) OR (#7) OR (#8) OR (#9) OR (#11) | 42,162 |
|  |  | "nonalcoholic fatty liver"[All Fields] OR "non-alcoholic fatty liver"[All Fields] OR ("non-alcoholic"[All Fields] AND "fatty liver"[All Fields]) OR "non-alcoholic fatty liver disease"[MeSH Terms] OR "NAFLD"[All Fields] OR "nonalcoholic steatohepatitis"[All Fields] OR "non-alcoholic steatohepatitis"[All Fields] OR "NASH"[All Fields] |  |
| Interventions and comparators | #14 | “Dipeptidyl-peptidase iv inhibitors” [MeSH] | 4,276 |
|  | #15 | “Dipeptidyl peptidase 4 inhibitors” [All Fields] | 1,505 |
|  | #16 | “DPP-4 inhibitors” [All Fields] | 1,994 |
|  | #17 | (#14) OR (#15) OR (#16) | 5,689 |
|  | #18 | “Alogliptin” [All Fields] | 548 |
|  | #19 | “Linagliptin” [All Fields] | 874 |
|  | #20 | “Sitagliptin” [All Fields] | 2,665 |
|  | #21 | “Vildagliptin” [All Fields] | 1,165 |
|  | #22 | “Trelagliptin” [All Fields] | 50 |
|  | #23 | (#14) OR (#15)) OR (#16) OR (#18) OR (#19) OR (#20) OR (#21) OR (#22) | 7,813 |
|  |  | "Dipeptidyl-peptidase iv inhibitors"[MeSH Terms] OR "Dipeptidyl peptidase 4 inhibitors"[All Fields] OR "DPP-4 inhibitors"[All Fields] OR "Alogliptin"[All Fields] OR "Linagliptin"[All Fields] OR "sitagliptin"[All Fields] OR "vildagliptin"[All Fields] OR "trelagliptin"[All Fields] |  |
|  | #24 | “glucagon-like peptide-1 receptor” [MeSH Terms] | 3,302 |
|  | #25 | “glucagon-like peptide 1” [All Fields] | 15,401 |
|  | #26 | “glucagon like peptide 1 receptor” [All Fields] | 5,074 |
|  | #27 | (#25) OR (#26) | 15,401 |
|  | #28 | “GLP-1RA” [All Fields] | 456 |
|  | #29 | (#27) OR (#28) | 15,452 |
|  | #30 | “liraglutide” [All Fields] | 3,273 |
|  | #31 | “exenatide” [All Fields] | 3,396 |
|  | #32 | “albiglutide” [All Fields] | 213 |
|  | #33 | “lixisenatide” [All Fields] | 500 |
|  | #34 | “dulaglutide” [All Fields] | 493 |
|  | #35 | “semaglutide” [All Fields] | 561 |
|  | #36 | (#24) OR (#25) OR (#28) OR (#30) OR (#31) OR (#32) OR (#33) OR (#34) OR (#35) | 17,890 |
|  |  | "glucagon-like peptide-1 receptor"[MeSH Terms] OR "glucagon-like peptide 1"[All Fields] OR "GLP-1RA"[All Fields] OR "liraglutide"[All Fields] OR "exenatide"[All Fields] OR "albiglutide"[All Fields] OR "lixisenatide"[All Fields] OR "dulaglutide"[All Fields] OR "semaglutide"[All Fields] |  |
|  | #37 | “SGLT2 inhibitors” [All Fields] | 2,158 |
|  | #38 | “sodium-glucose cotransporter-2 inhibitors” [All Fields] | 1,079 |
|  | #39 | (#37) OR (#38) | 2,907 |
|  | #40 | “empagliflozin” [All Fields] | 1,710 |
|  | #41 | “canagliflozin” [All Fields] | 1,352 |
|  | #42 | “Luseogliflozin” [All Fields] | 106 |
|  | #43 | “Dapagliflozin” [All Fields] | 1,657 |
|  | #44 | “Ertugliflozin” [All Fields] | 152 |
|  | #45 | “Ipragliflozin” [All Fields] | 247 |
|  | #46 | “Tofogliflozin” [All Fields] | 123 |
|  | #47 | (#37) OR (#38) OR (#40) OR (#41) OR (#42) OR (#43) OR (#44) OR (#45) OR (#46) | 5,698 |
|  |  | "SGLT2 inhibitors"[All Fields] OR "sodium-glucose cotransporter-2 inhibitors"[All Fields] OR "empagliflozin"[All Fields] OR "Tofogliflozin"[All Fields] OR "canagliflozin"[All Fields] OR "Luseogliflozin"[All Fields] OR "Dapagliflozin"[All Fields] OR "Ertugliflozin"[All Fields] OR "Ipragliflozin"[All Fields] |  |
| Patients, interventions and comparators | #48 | (#13) AND ((#23) OR (#36) OR (#47)) | 22,944 |
|  | #49 | #48 filter systematic review and meta-analysis | 709 |
|  | #50 | #49 filter randomized controlled trials limit 2020-2021 | 110 |

- **Search strategy in Scopus databased (search date 3^rd^ June 2021)**

| **Domains** | **Search number** | **Search term** | **Results (studies)** |
| --- | --- | --- | --- |
| Population | #1 | TITLE-ABS-KEY: "nonalcoholic fatty liver" | 32,617 |
|  | #2 | TITLE-ABS-KEY: "non-alcoholic fatty liver" | 19,789 |
|  | #3 | TITLE-ABS-KEY: "non-alcoholic" AND "fatty liver" | 21,418 |
|  | #4 | TITLE-ABS-KEY: "non-alcoholic fatty liver disease" | 19,435 |
|  | #5 | TITLE-ABS-KEY: "NAFLD" | 18,220 |
|  | #6 | TITLE-ABS-KEY: "nonalcoholic steatohepatitis" | 8,130 |
|  | #7 | TITLE-ABS-KEY: “non-alcoholic steatohepatitis” | 5,244 |
|  | #8 | TITLE-ABS-KEY: “NASH” | 43,916 |
|  | #9 | (#1) OR (#2) OR (#3) OR (#4) OR (#5) OR (#6) OR (#7) OR (#8) | 74,436 |
|  |  | "nonalcoholic fatty liver" OR "non-alcoholic fatty liver" OR ("non-alcoholic" AND "fatty liver") OR "non-alcoholic fatty liver disease" OR "NAFLD” OR "nonalcoholic steatohepatitis" OR "non-alcoholic steatohepatitis" OR "NASH" |  |
| Interventions and comparators | #10 | TITLE-ABS-KEY: “Dipeptidyl-peptidase iv inhibitors” | 14,603 |
|  | #11 | TITLE-ABS-KEY: “Dipeptidyl peptidase 4 inhibitors” | 13,891 |
|  | #12 | TITLE-ABS-KEY: “DPP-4 inhibitors” | 9,842 |
|  | #13 | TITLE-ABS-KEY: “Alogliptin” | 5,025 |
|  | #14 | TITLE-ABS-KEY: “Linagliptin” | 5,719 |
|  | #15 | TITLE-ABS-KEY: “Sitagliptin” | 19,910 |
|  | #16 | TITLE-ABS-KEY: “Vildagliptin” | 8,293 |
|  | #17 | TITLE-ABS-KEY: “Trelagliptin” | 239 |
|  | #18 | (#10) OR (#11) OR (#12) OR (#13) OR (#14) OR (#15) OR (#16) OR (#17) | 37,463 |
|  |  | "Dipeptidyl-peptidase iv inhibitors” OR "Dipeptidyl peptidase 4 inhibitors" OR "DPP-4 inhibitors" OR "Alogliptin" OR "Linagliptin" OR "sitagliptin" OR "vildagliptin" OR "trelagliptin" |  |
|  | #19 | TITLE-ABS-KEY: “glucagon-like peptide-1 receptor” | 21,643 |
|  | #20 | TITLE-ABS-KEY: “glucagon-like peptide 1” | 55,647 |
|  | #21 | TITLE-ABS-KEY: “GLP-1RA” | 573 |
|  | #22 | TITLE-ABS-KEY: “liraglutide” | 19,560 |
|  | #23 | TITLE-ABS-KEY: “exenatide” | 15,558 |
|  | #24 | TITLE-ABS-KEY: “albiglutide” | 2,081 |
|  | #25 | TITLE-ABS-KEY: “lixisenatide” | 3,522 |
|  | #26 | TITLE-ABS-KEY: “dulaglutide” | 2,658 |
|  | #27 | TITLE-ABS-KEY: “semaglutide” | 3,534 |
|  | #28 | (#19) OR (#20) OR (#21) OR (#22) OR (#23) OR (#24) OR (#25) OR (#26) OR (#27) | 67,948 |
|  |  | "glucagon-like peptide-1 receptor" OR "glucagon-like peptide 1" OR "GLP-1RA" OR "liraglutide" OR "exenatide” OR "albiglutide" OR "lixisenatide" OR "dulaglutide" OR "semaglutide" |  |
|  | #29 | TITLE-ABS-KEY: “SGLT2 inhibitors” | 3,824 |
|  | #30 | TITLE-ABS-KEY: “sodium-glucose cotransporter-2 inhibitors” | 6,644 |
|  | #31 | TITLE-ABS-KEY: “empagliflozin” | 3,533 |
|  | #32 | TITLE-ABS-KEY: “canagliflozin” | 2,870 |
|  | #33 | TITLE-ABS-KEY: “Luseogliflozin” | 293 |
|  | #34 | TITLE-ABS-KEY: “Dapagliflozin” | 3,466 |
|  | #35 | TITLE-ABS-KEY: “Ertugliflozin” | 462 |
|  | #36 | TITLE-ABS-KEY: “Ipragliflozin” | 523 |
|  | #37 | TITLE-ABS-KEY: “Tofogliflozin” | 300 |
|  | #38 | (#29) OR (#30) OR (#31) OR (#32) OR (#33) OR (#34) OR (#35) OR (#36) OR (#37) | 10,643 |
|  |  | "SGLT2 inhibitors” OR "sodium-glucose cotransporter-2 inhibitors” OR "empagliflozin” OR "Tofogliflozin" OR "Canagliflozin” OR "Luseogliflozin" OR "Dapagliflozin" OR "Ertugliflozin OR "Ipragliflozin” |  |
| Patients, interventions and comparators | #39 | (#9) AND ((#18) OR (#28) OR (#38)) | 2,769 |
|  | #40 | #39 AND systematic review OR meta-analysis | 220 |
|  | #41 | #39 AND Randomized controlled trials with limit Year 2020-2021 | 87 |

**Supplementary Table S2.** SUCRA (%) for the efficacy of each treatment on intrahepatic steatosis, liver enzymes, body mass index (BMI) and adverse events in T2DM patients with NAFLD.

| Treatment | Intrahepatic steatosis | | Liver enzymes | | | BMI | Adverse event |
| --- | --- | --- | --- | --- | --- | --- | --- |
|  |  |  | **AST** | **ALT** | **GGT** |  |  |
| SoC | | 21.5 | 43.5 | 44.8 | 47.6 | 47.5 | 54.1 |
| Omega-3 | | 6.4 | 6.5 | 10.7 | 12.3 | - | 38.8 |
| Metformin | | - | 35.2 | 47.1 | 84.5 | 61.4 | 46.9 |
| Sulfonylurea | | - | 25.2 | 29.9 | 11.3 | 25.1 | 98.7 |
| Pioglitazone | | 62.2 | 67.3 | 77.0 | 48.6 | 20.1 | 58.2 |
| Insulin | | 57.8 | 34.1 | 46.2 | 74.6 | 34.6 | 23.9 |
| DPP-4 inhibitors | | 69.6 | 68.8 | 39.8 | 32.8 | 51.5 | 50.6 |
| GLP-1 agonists | | 88.5 | 95.5 | 97.2 | 99.6 | 95.3 | 5.3 |
| SGLT-2 inhibitors | | 44.1 | 74.0 | 57.3 | 38.6 | 64.5 | 73.6 |

**Supplementary Table S3**. Relative treatment effects by mean difference on GGT: A network meta-analysis.

| SoC | 0.45  (-4.00,4.91) | -15.53  (-22.09,-8.97) | 0.85  (-0.31,2.01) | -6.25  (-13.82,1.31) | -1.09  (-7.61,5.43) | 12.05  (-3.31,27.41) | -9.61  (-18.28,-0.94) | 19.07  (-11.93,50.06) |
| --- | --- | --- | --- | --- | --- | --- | --- | --- |
| -0.45  (-4.91,4.00) | **SGLT-2is** | **-15.98**  **(-21.12,-10.85)** | 0.40  (-4.20,5.00) | -6.71  (-13.08,-0.33) | -1.54  (-6.63,3.54) | 11.60  (-3.16,26.37) | **-10.06**  **(-17.72,-2.41)** | 18.61  (-12.61,49.84) |
| 15.53  (8.97,22.09) | **15.98**  **(10.85,21.12**) | **GLP-1 RAs** | 16.38 (9.72,23.04) | 9.27 (5.50,13.05) | 14.44 (13.64,15.23) | 27.58 (12.85,42.32) | 5.92 (0.24,11.60) | 34.60 (2.99,66.20) |
| -0.85  (-2.01,0.31) | -0.40  (-5.00,4.20) | -16.38  (-23.04,-9.72) | **DPP-4is** | -7.11  (-14.76,0.55) | -1.94  (-8.57,4.68) | 11.20  (-4.20,26.61) | **-10.46**  **(-19.21,-1.71**) | 18.21  (-12.80,49.23) |
| 6.25  (-1.31,13.82) | 6.71 (0.33,13.08) | -9.27  (-13.05,-5.50) | 7.11  (-0.55,14.76) | **Insulin** | 5.16 (1.30,9.02) | 18.31 (3.10,33.52) | -3.36  (-10.18,3.46) | 25.32  (-6.51,57.15) |
| 1.09  (-5.43,7.61) | 1.54  (-3.54,6.63) | -14.44  (-15.23,-13.64) | 1.94  (-4.68,8.57) | -5.16  (-9.02,-1.30) | **Pioglitazone** | 13.14  (-1.57,27.86) | **-8.52**  **(-14.25,-2.78)** | 20.16  (-11.44,51.75) |
| -12.05  (-27.41,3.31) | -11.60  (-26.37,3.16) | -27.58  (-42.32,-12.85) | -11.20  (-26.61,4.20) | -18.31  (-33.52,-3.10) | -13.14  (-27.86,1.57) | **Sulfonylurea** | **-21.66**  **(-37.45,-5.88)** | 7.01  (-27.50,41.53) |
| 9.61 (0.94,18.28) | **10.06 (2.41,17.72)** | -5.92  (-11.60,-0.24) | **10.46 (1.71,19.21)** | 3.36  (-3.46,10.18) | **8.52 (2.78,14.25)** | **21.66**  **(5.88,37.45)** | **Metformin** | 28.68  (-3.43,60.78) |
| -19.07  (-50.06,11.93) | -18.61  (-49.84,12.61) | -34.60  (-66.20,-2.99) | -18.21  (-49.23,12.80) | -25.32  (-57.15,6.51) | -20.16  (-51.75,11.44) | -7.01  (-41.53,27.50) | -28.68  (-60.78,3.43) | **Omega-3** |

SoC: standard of care, SGLT-2is: SGLT-2 inhibitors, GLP-1 RAs: GLP-1 receptor agonists, DPP-4is: DPP-4 inhibitors

**Supplementary Table S4.** Relative treatment effects by mean difference on body mass index (BMI): A network meta-analysis.

| SoC | -0.38  (-1.64,0.89) | -1.30  (-2.92,0.32) | -0.07  (-2.19,2.05) | 0.37  (-1.57,2.31) | 0.93  (-1.03,2.89) | 0.90  (-1.81,3.61) | -0.27  (-1.98,1.44) |
| --- | --- | --- | --- | --- | --- | --- | --- |
| 0.38  (-0.89,1.64) | **SGLT-2is** | -0.92  (-2.56,0.71) | 0.31  (-1.93,2.56) | 0.75  (-1.21,2.71) | 1.31  (-0.62,3.23) | 1.28  (-1.41,3.97) | 0.11  (-1.52,1.74) |
| 1.30  (-0.32,2.92) | 0.92  (-0.71,2.56) | **GLP-1 RAs** | 1.24  (-0.67,3.14) | 1.67 (0.47,2.87) | 2.23 (0.31,4.15) | 2.20  (-0.04,4.44) | 1.03  (-0.00,2.06) |
| 0.07  (-2.05,2.19) | -0.31  (-2.56,1.93) | -1.24  (-3.14,0.67) | **DPP-4is** | 0.44  (-1.61,2.48) | 0.99  (-1.55,3.54) | 0.97  (-1.95,3.89) | -0.20  (-2.30,1.89) |
| -0.37  (-2.31,1.57) | -0.75  (-2.71,1.21) | **-1.67**  **(-2.87,-0.47)** | -0.44  (-2.48,1.61) | **Insulin** | 0.56  (-1.67,2.79) | 0.53  (-2.00,3.05) | -0.64  (-2.12,0.83) |
| -0.93  (-2.89,1.03) | -1.31  (-3.23,0.62) | **-2.23**  **(-4.15,-0.31)** | -0.99  (-3.54,1.55) | -0.56  (-2.79,1.67) | **Pioglitazone** | -0.03  (-2.94,2.88) | -1.20  (-3.25,0.85) |
| -0.90  (-3.61,1.81) | -1.28  (-3.97,1.41) | -2.20  (-4.44,0.04) | -0.97  (-3.89,1.95) | -0.53  (-3.05,2.00) | 0.03  (-2.88,2.94) | **Sulfonylurea** | -1.17  (-3.45,1.11) |
| 0.27  (-1.44,1.98) | -0.11  (-1.74,1.52) | -1.03  (-2.06,0.00) | 0.20  (-1.89,2.30) | 0.64  (-0.83,2.12) | 1.20  (-0.85,3.25) | 1.17  (-1.11,3.45) | **Metformin** |

SoC: standard of care, SGLT-2is: SGLT-2 inhibitors, GLP-1 RAs: GLP-1 receptor agonists, DPP-4is: DPP-4 inhibitors

**Supplementary Table S5**. Relative treatment effects by risk difference on any adverse event: A network meta-analysis.

| **SoC** | -0.05  (-0.17,0.06) | **0.17 (0.06,0.28)** | 0.01  (-0.13,0.15) | 0.09  (-0.05,0.24) | -0.02  (-0.14,0.11) | **-0.22**  **(-0.39,-0.05)** | 0.02  (-0.15,0.20) | 0.07  (-0.24,0.37) |
| --- | --- | --- | --- | --- | --- | --- | --- | --- |
| 0.05  (-0.06,0.17) | **SGLT-2is** | **0.22 (0.08,0.36)** | 0.06  (-0.11,0.23) | 0.15  (-0.03,0.32) | 0.04  (-0.07,0.14) | **-0.17**  **(-0.33,-0.00)** | 0.07  (-0.12,0.26) | 0.12  (-0.19,0.42) |
| **-0.17**  **(-0.28,-0.06)** | **-0.22**  **(-0.36,-0.08)** | **GLP-1 RAs** | **-0.16**  **(-0.30,-0.02)** | -0.08  (-0.21,0.06) | **-0.19**  **(-0.33,-0.04)** | **-0.39**  **(-0.55,-0.22)** | -0.15  (-0.30,0.01) | -0.10  (-0.42,0.22) |
| -0.01  (-0.15,0.13) | -0.06  (-0.23,0.11) | **0.16 (0.02,0.30)** | **DPP-4is** | 0.08  (-0.08,0.25) | -0.03  (-0.20,0.15) | **-0.23**  **(-0.43,-0.02)** | 0.01  (-0.18,0.20) | 0.06  (-0.28,0.39) |
| -0.09  (-0.24,0.05) | -0.15  (-0.32,0.03) | 0.08  (-0.06,0.21) | -0.08  (-0.25,0.08) | **Insulin** | -0.11  (-0.29,0.07) | **-0.31**  **(-0.52,-0.11)** | -0.07  (-0.27,0.12) | -0.03  (-0.36,0.31) |
| 0.02  (-0.11,0.14) | -0.04  (-0.14,0.07) | **0.19 (0.04,0.33)** | 0.03  (-0.15,0.20) | 0.11  (-0.07,0.29) | **Pioglitazone** | **-0.20**  **(-0.37,-0.03)** | 0.04  (-0.16,0.23) | 0.08  (-0.24,0.40) |
| **0.22 (0.05,0.39)** | **0.17 (0.00,0.33)** | **0.39 (0.22,0.55)** | **0.23 (0.02,0.43)** | **0.31 (0.11,0.52)** | **0.20 (0.03,0.37)** | **Sulfonylurea** | **0.24 (0.04,0.43)** | 0.28  (-0.06,0.62) |
| -0.02  (-0.20,0.15) | -0.07  (-0.26,0.12) | 0.15  (-0.01,0.30) | -0.01  (-0.20,0.18) | 0.07  (-0.12,0.27) | -0.04  (-0.23,0.16) | **-0.24**  **(-0.43,-0.04)** | **Metformin** | 0.05  (-0.30,0.39) |
| -0.07  (-0.37,0.24) | -0.12  (-0.42,0.19) | 0.10  (-0.22,0.42) | -0.06  (-0.39,0.28) | 0.03  (-0.31,0.36) | -0.08  (-0.40,0.24) | -0.28  (-0.62,0.06) | -0.05  (-0.39,0.30) | **Omega-3** |

SoC: standard of care, SGLT-2is: SGLT-2 inhibitors, GLP-1 RAs: GLP-1 receptor agonists, DPP-4is: DPP-4 inhibitors

**Supplementary Figure S1:** Forrest plots of pairwise meta-analysis

1. **Intrahepatic steatosis evaluated by *MRI liver.***

- ***GLP-1 analog VS insulin***


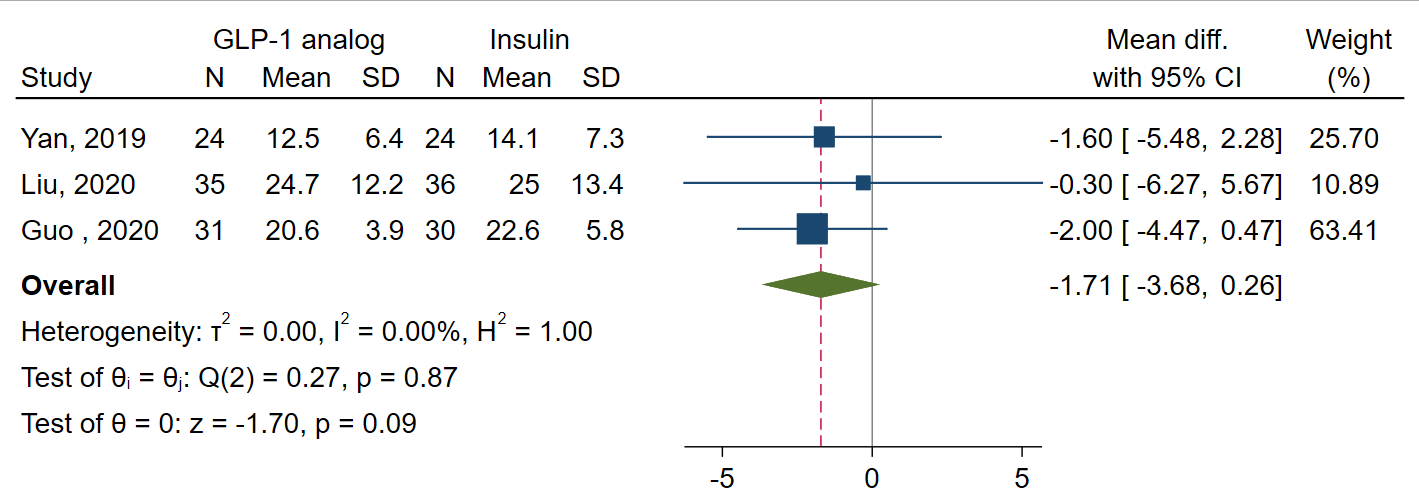


1. ***AST***

- ***GLP-1 analog VS insulin***


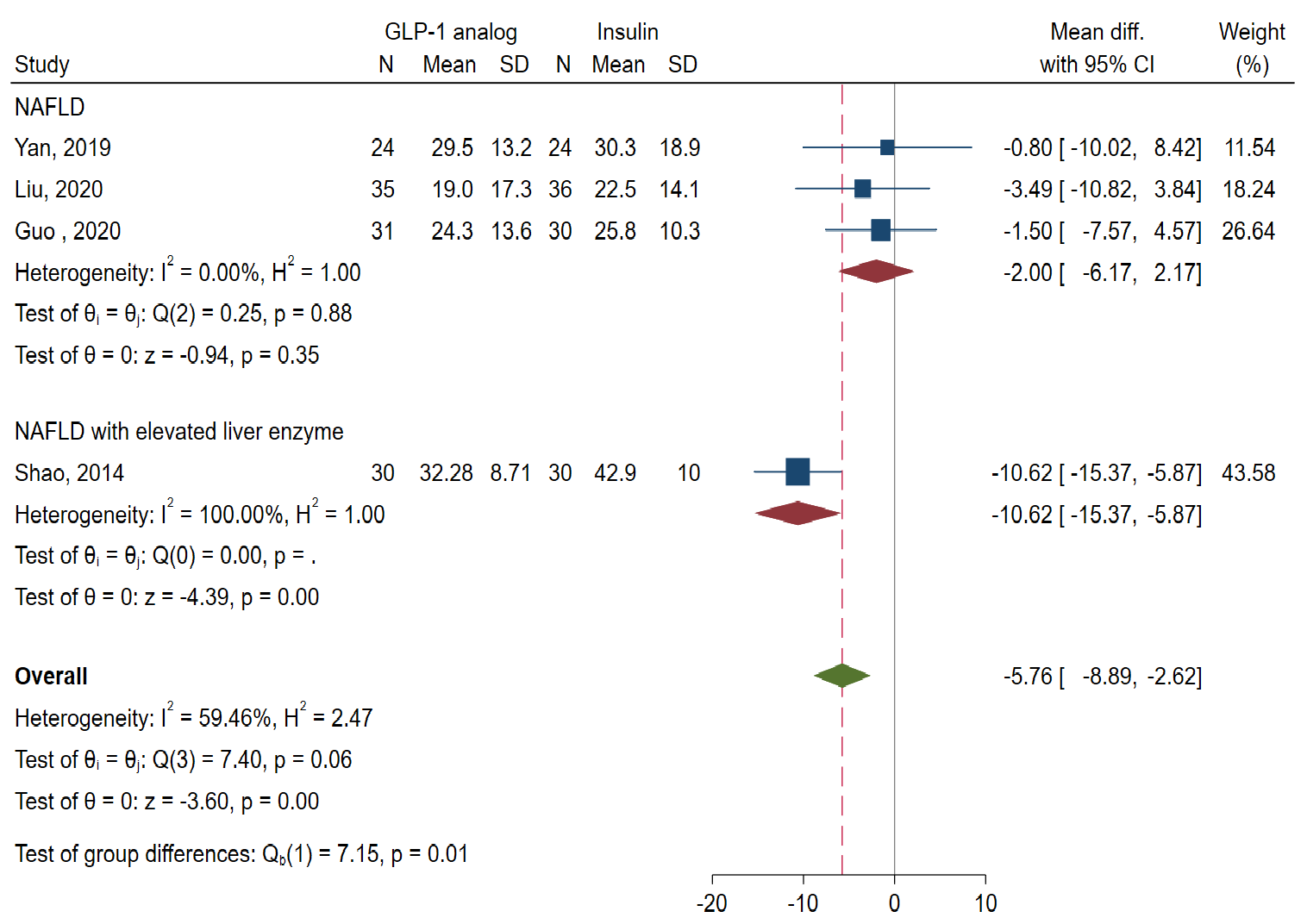


- ***GLP-1 analog VS metformin***


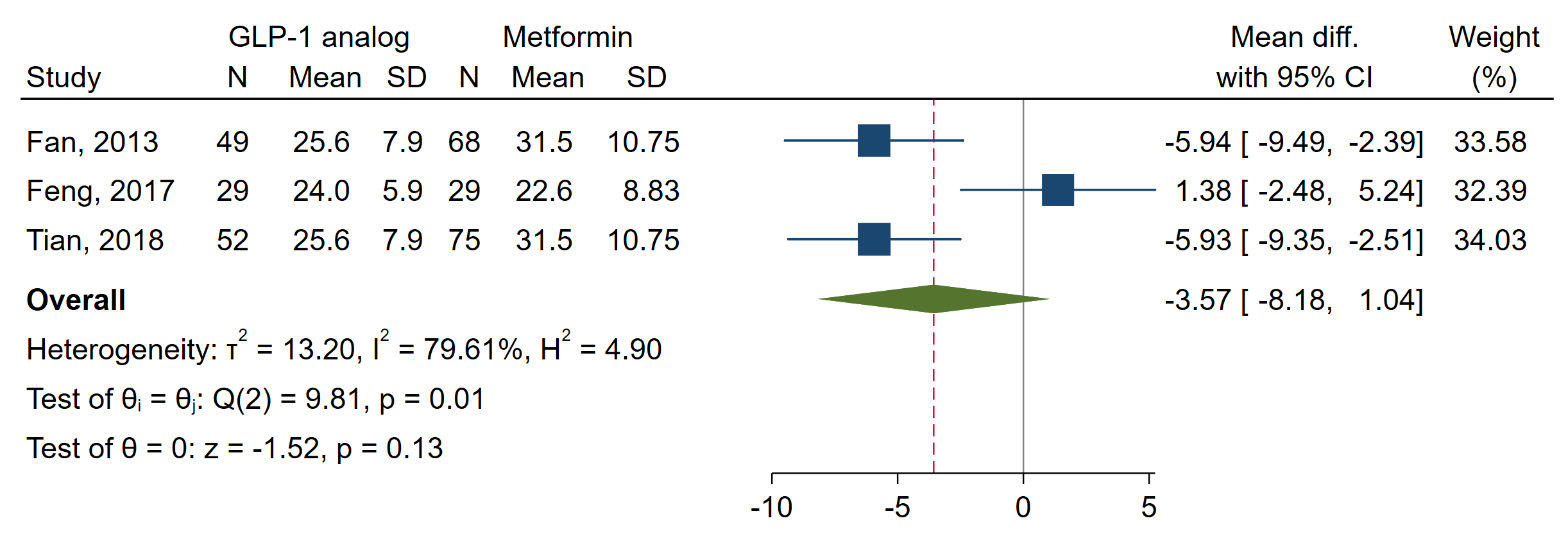


- ***SGLT-2 inhibitor VS pioglitazone***


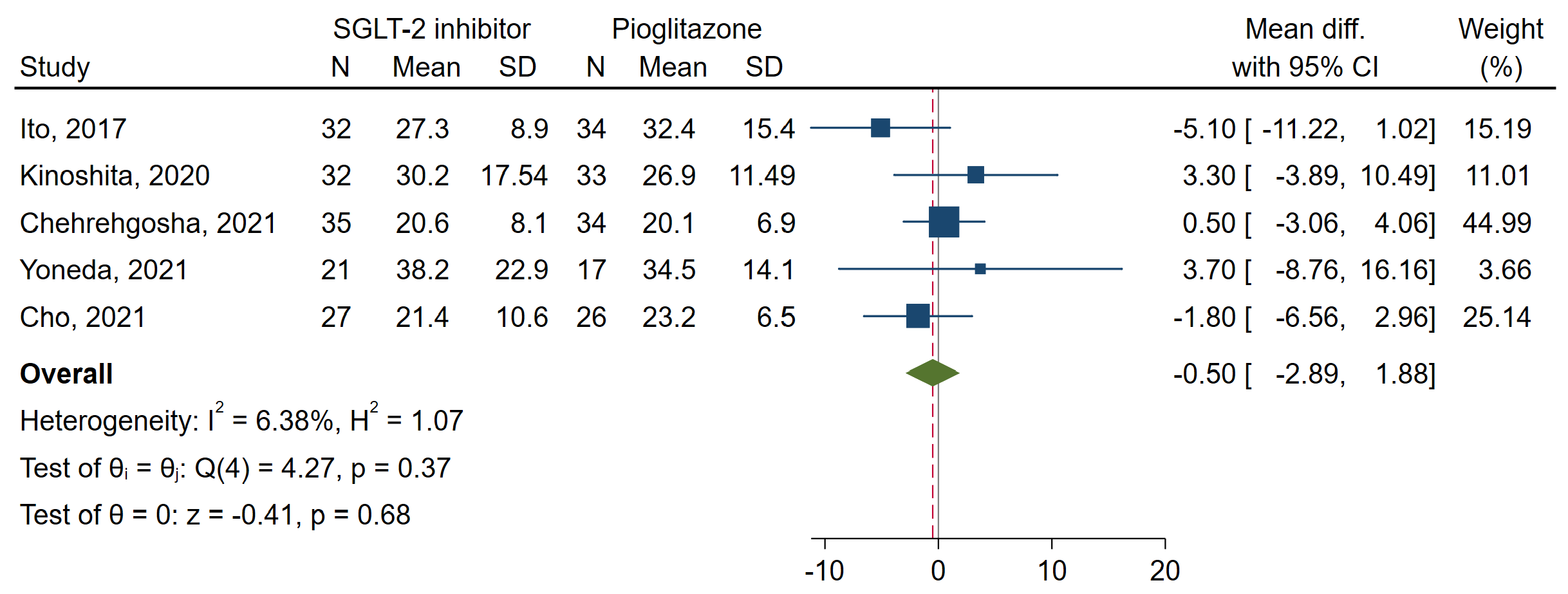


- ***SGLT-2 inhibitor VS standard of care***


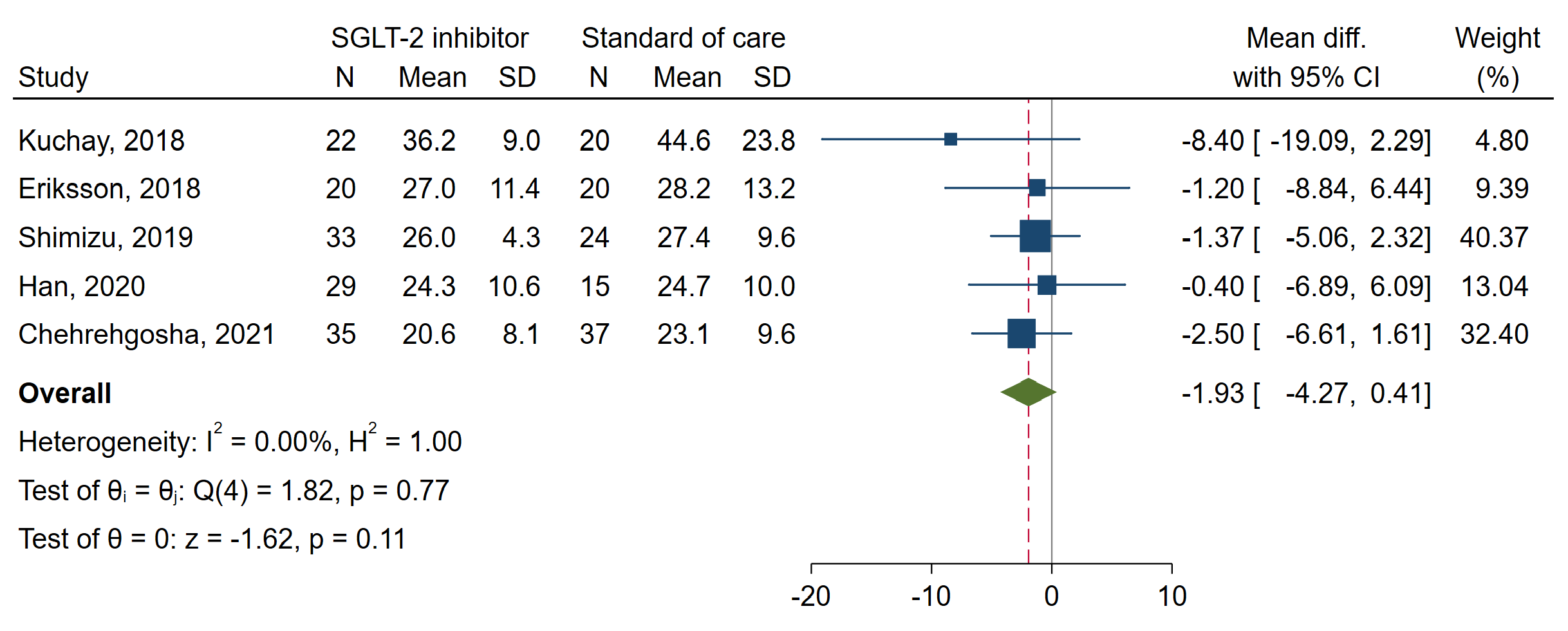


1. ***ALT***

- ***GLP-1 agonist VS insulin***
-
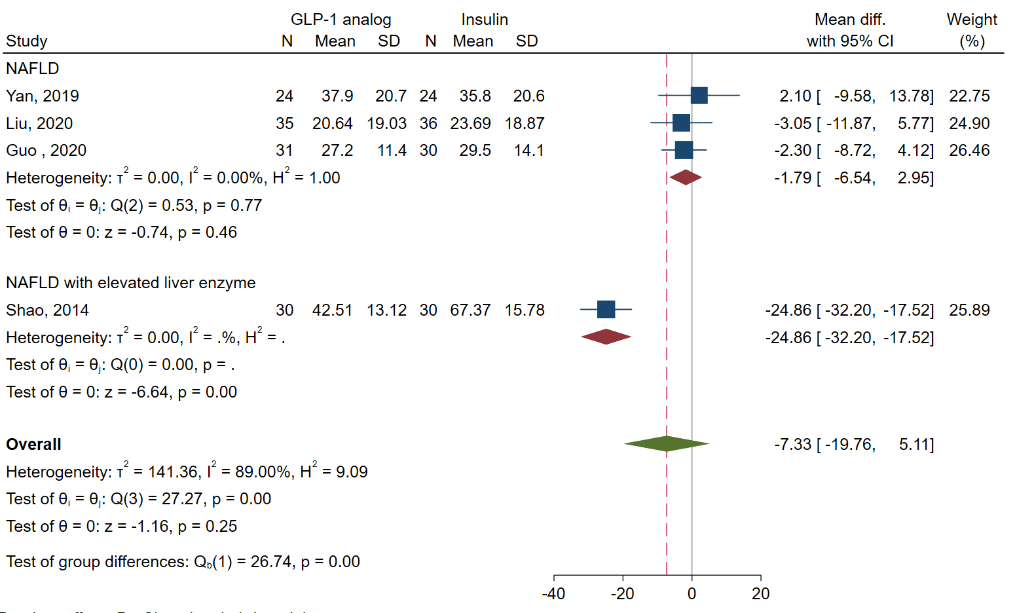

- ***GLP-1 agonist VS metformin***


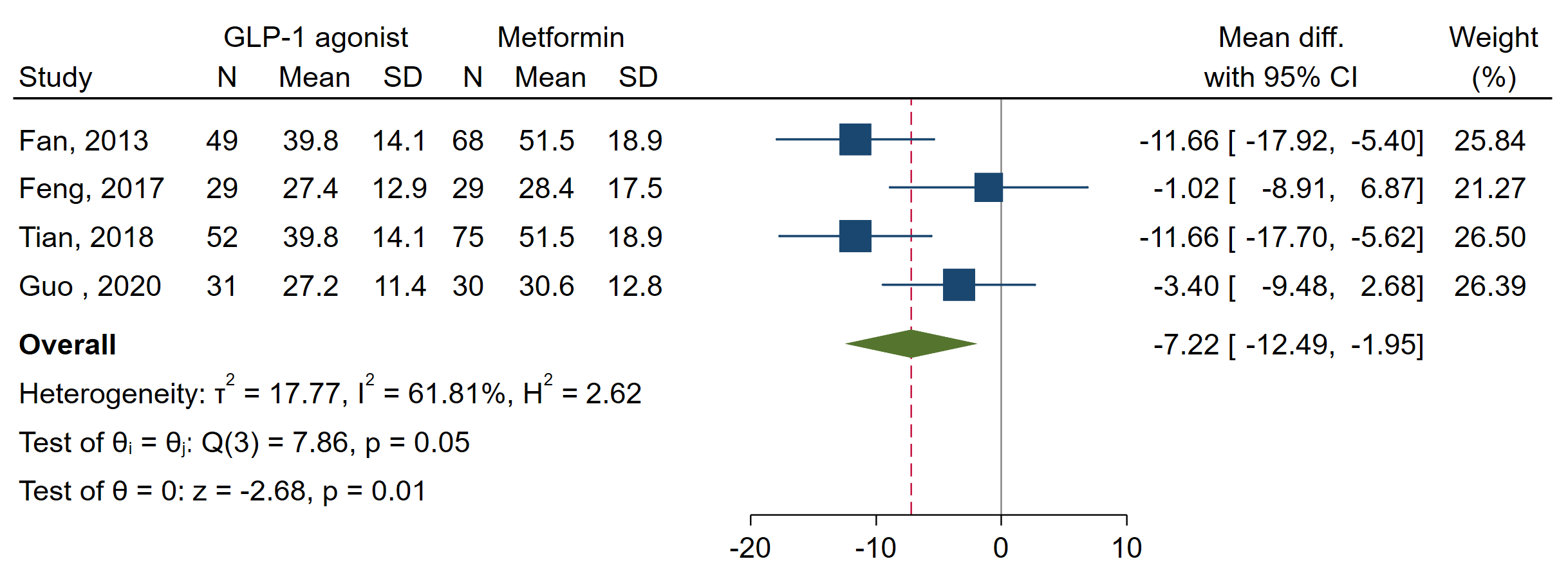


- ***SGLT-2 inhibitor VS pioglitazone***


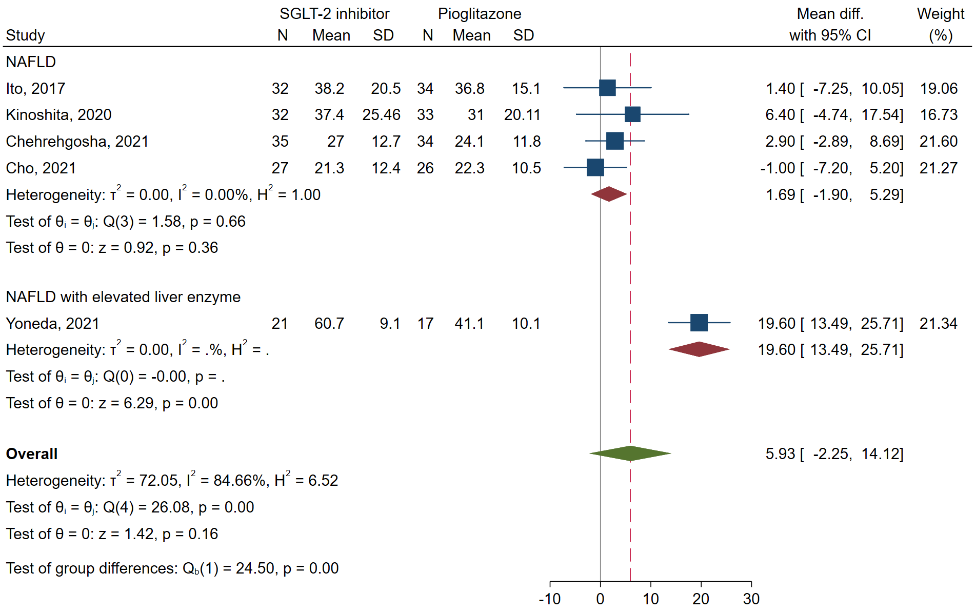


- ***SGLT-2 inhibitor VS standard of care***


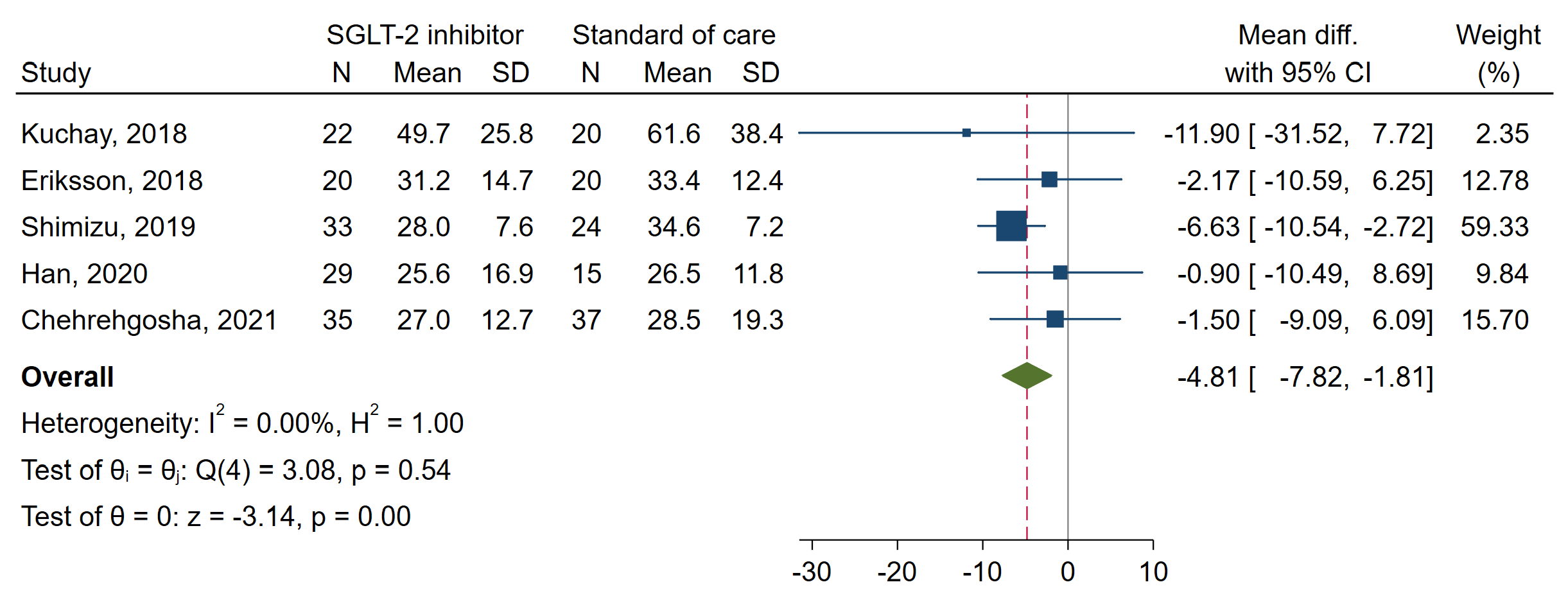


1. **GGT**

- ***GLP-1 agonist VS insulin***


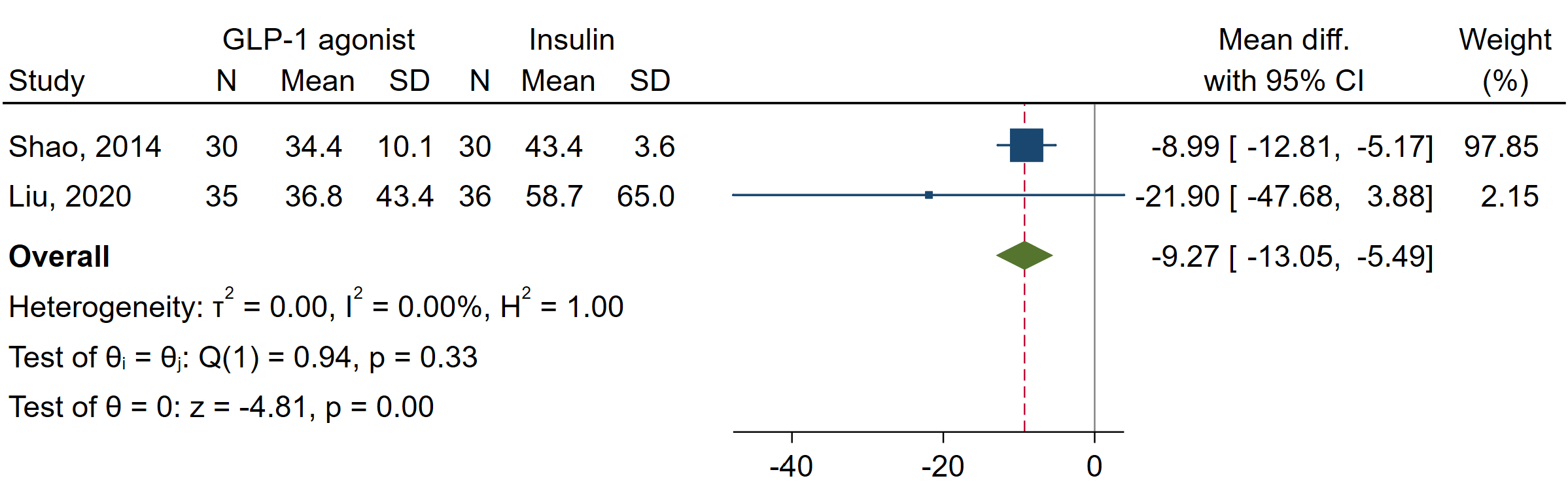


- ***SGLT-2 inhibitor VS pioglitazone***

**
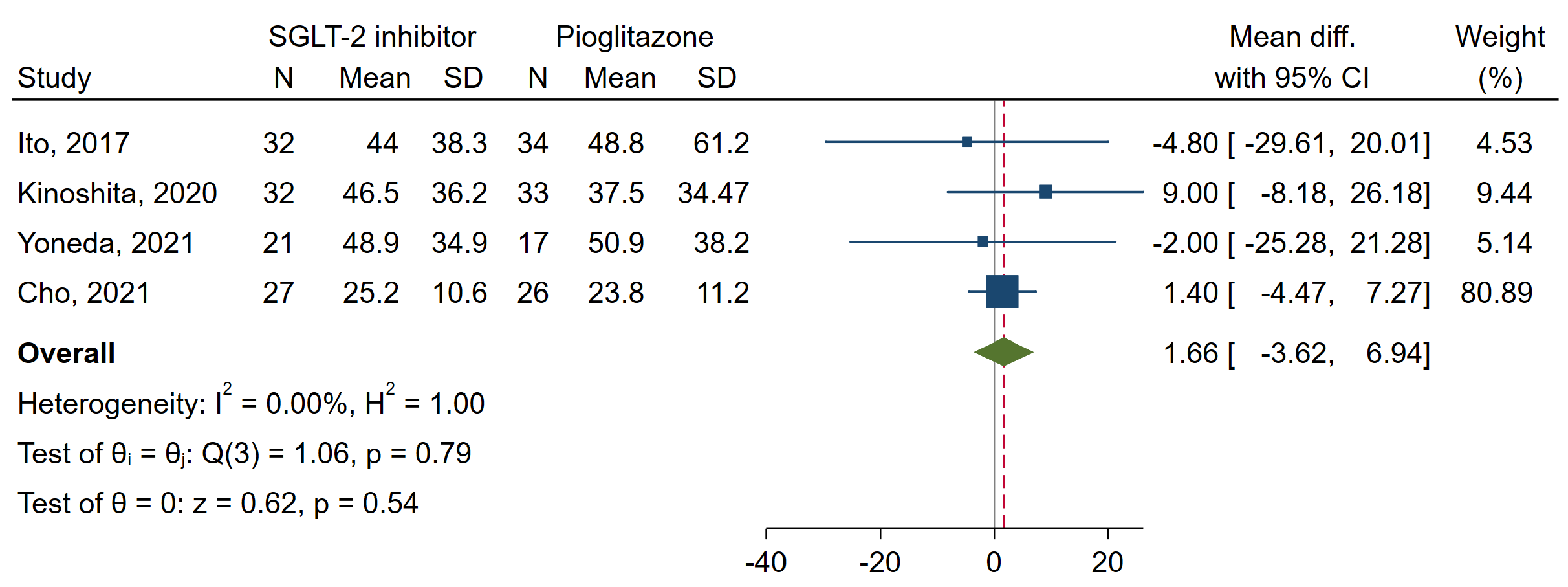
**

- ***SGLT-2 inhibitor VS standard of care***

**
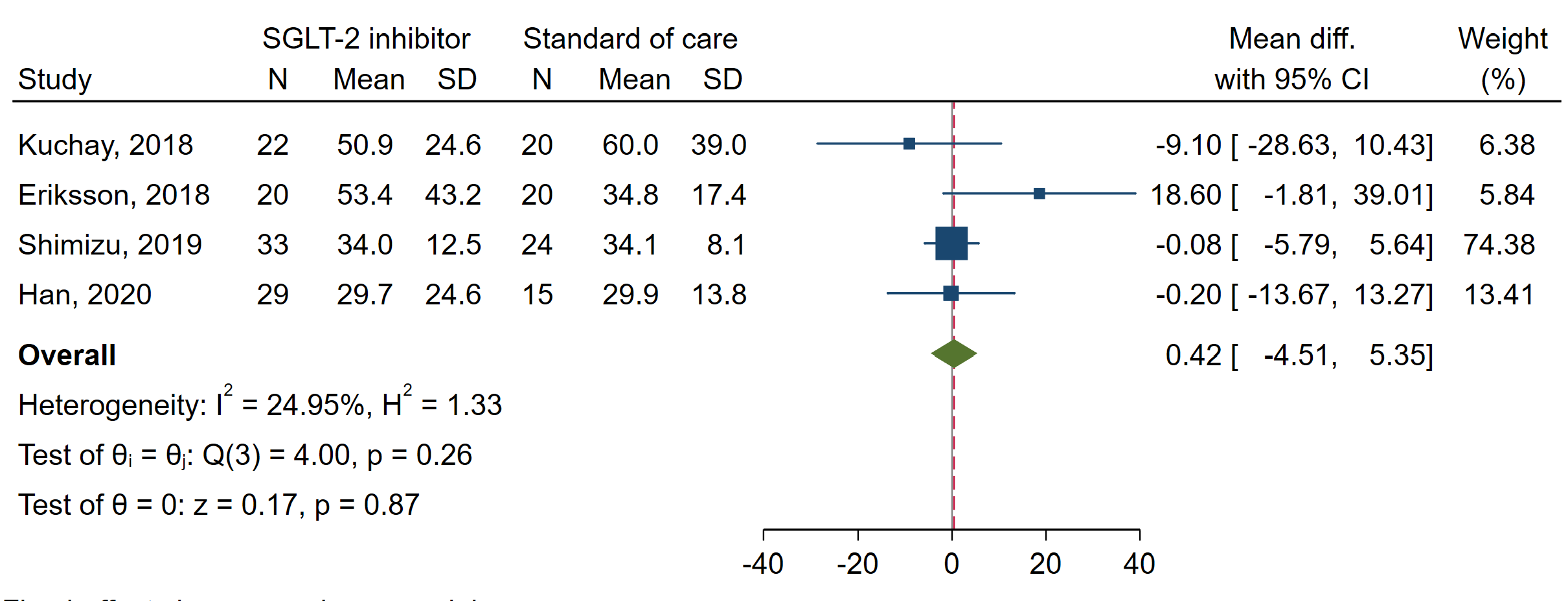
**

1. **BMI**

- ***GLP-1 agonist VS insulin***

**
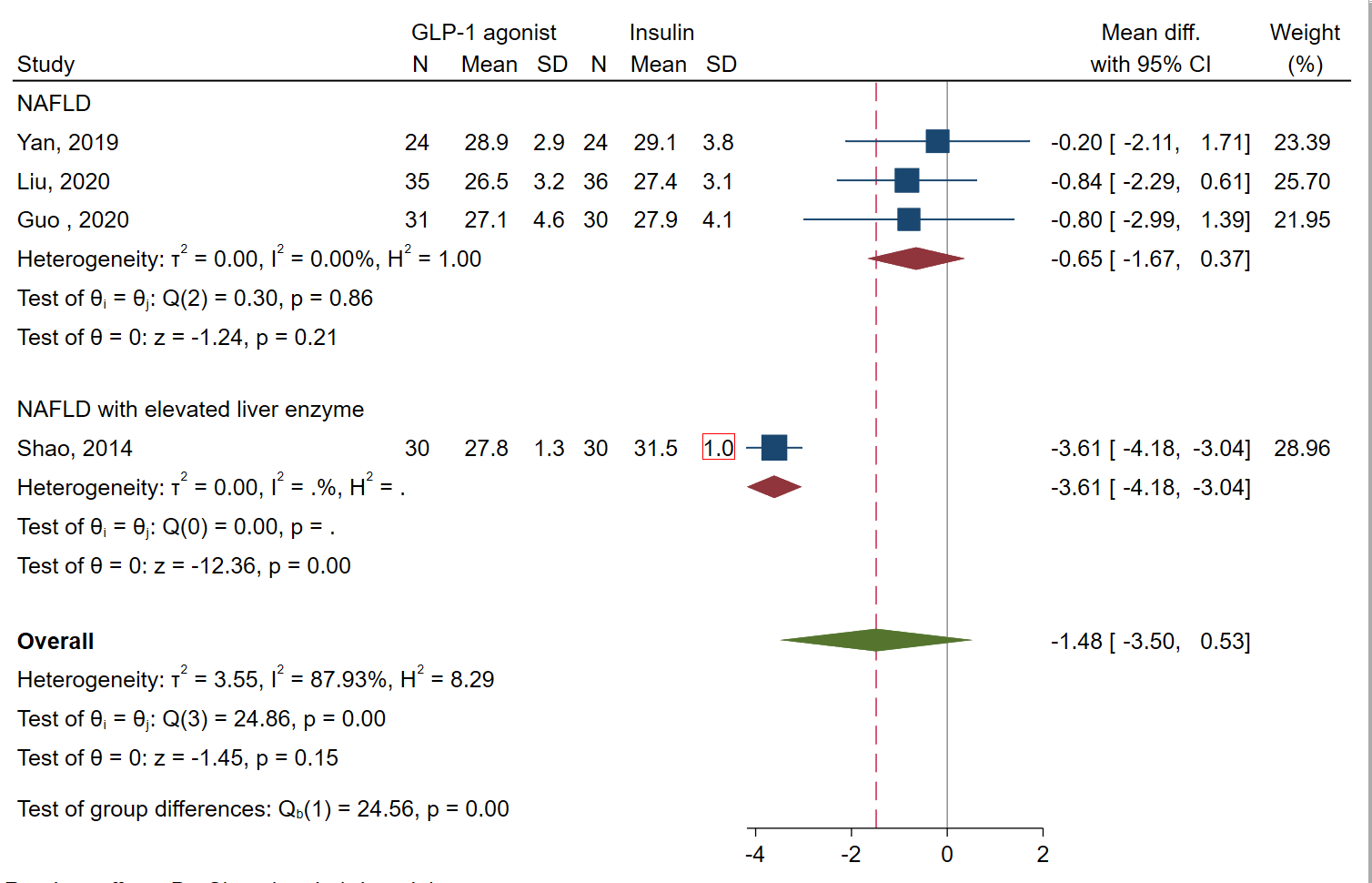
**

- ***GLP-1 agonist VS metformin***

**
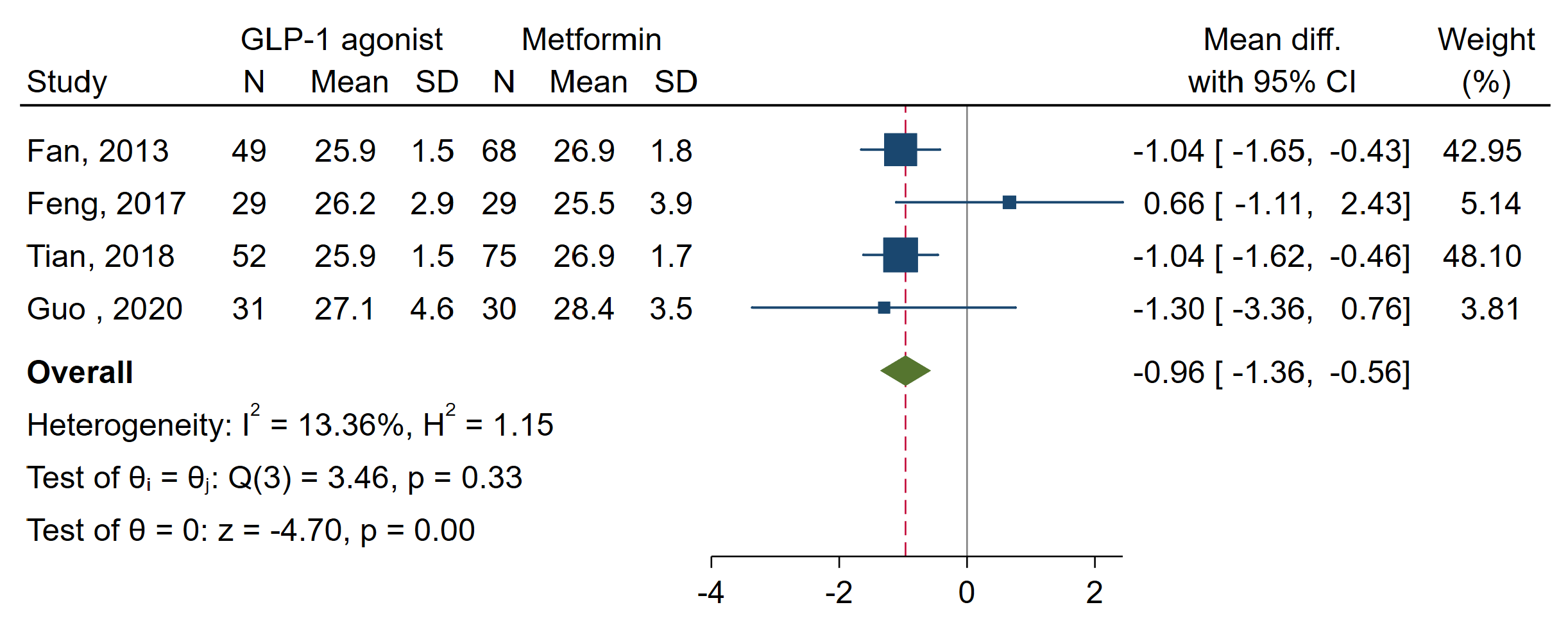
**

- ***SGLT-2 inhibitor VS standard of care***

**
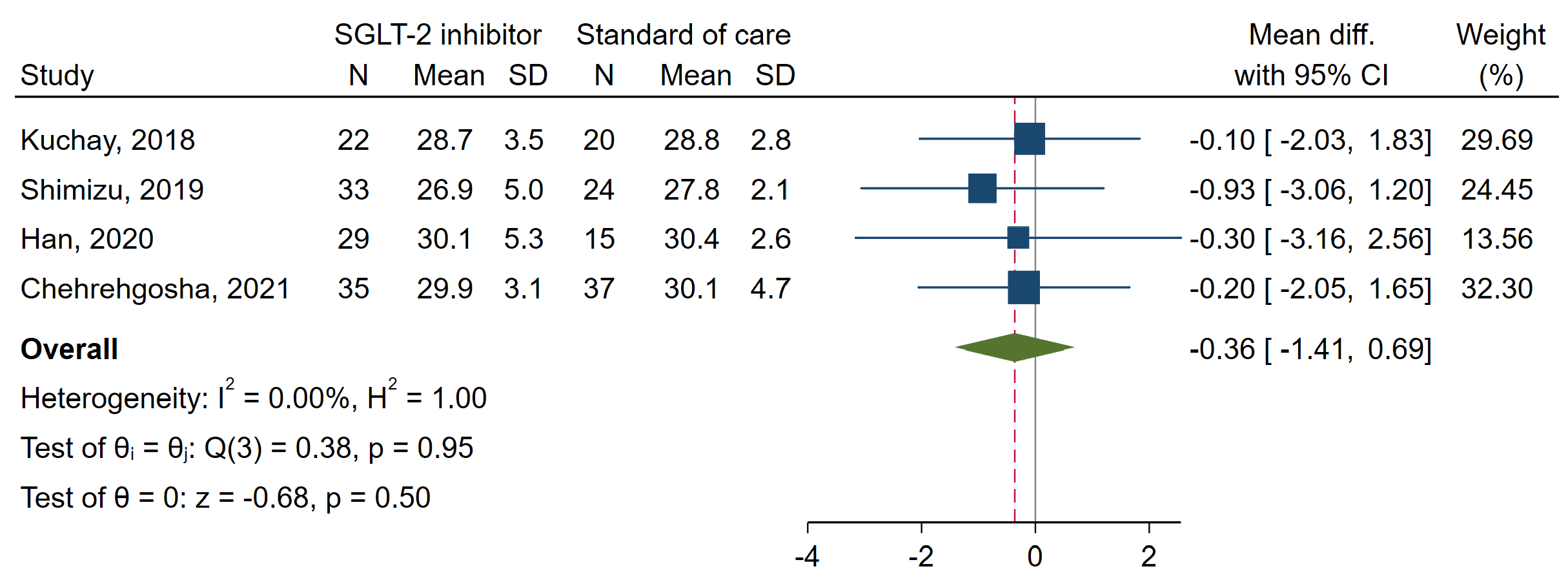
**

1. **Any adverse events**

- ***GLP-1 agonist VS Standard of care***


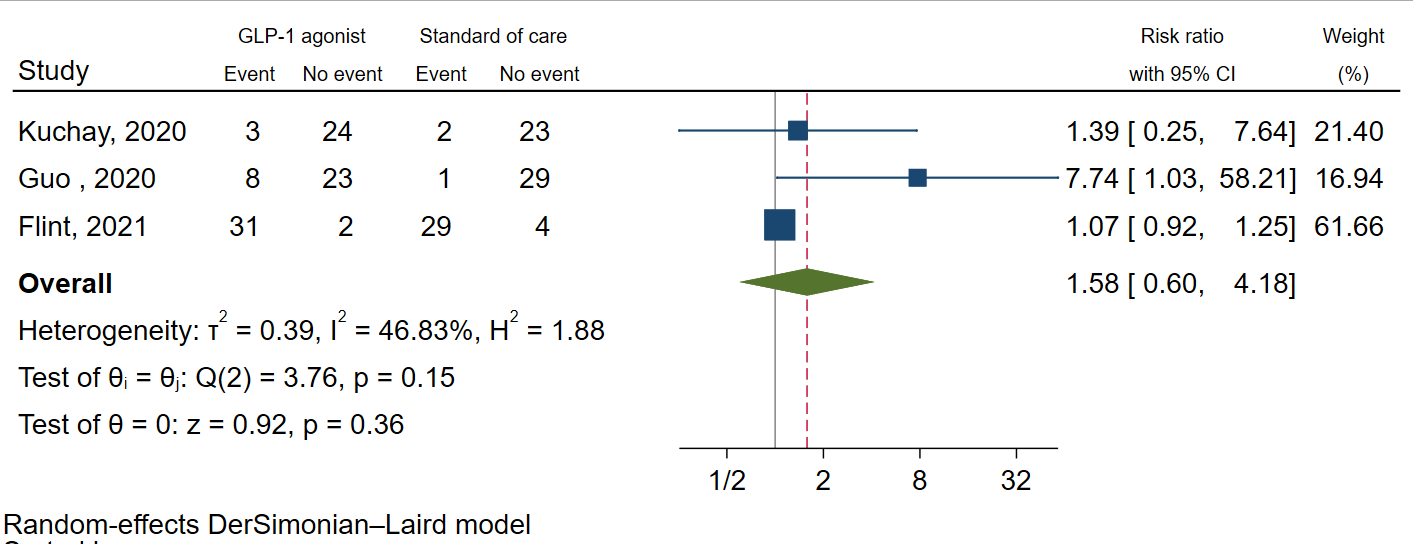


- ***SGLT-2 inhibitor VS Standard of care***

***
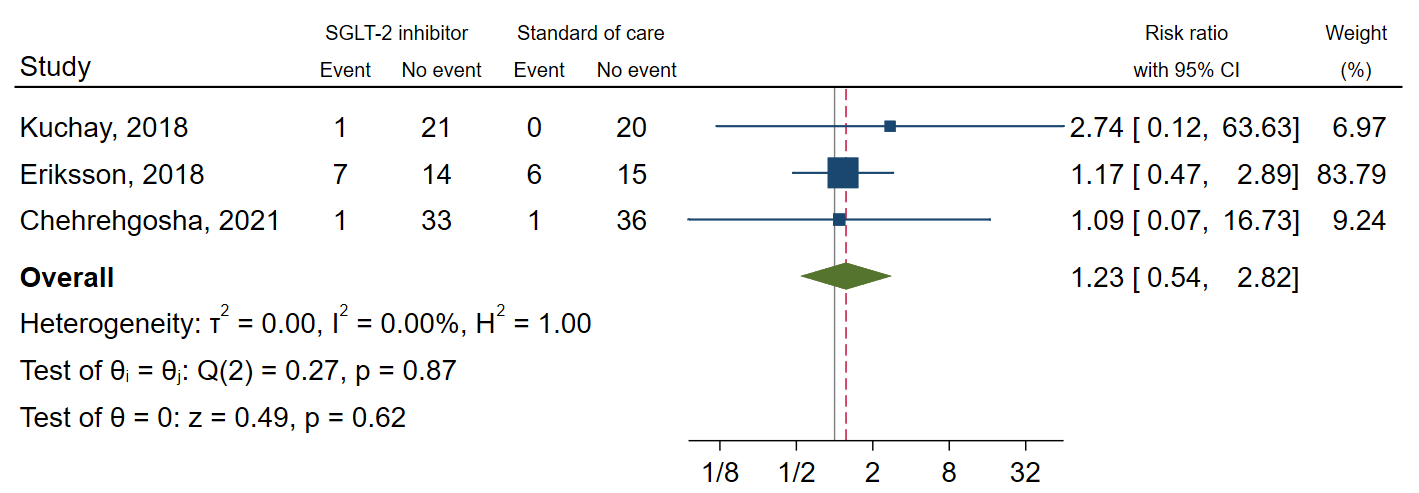
***

- ***SGLT-2 inhibitor VS Pioglitazone***


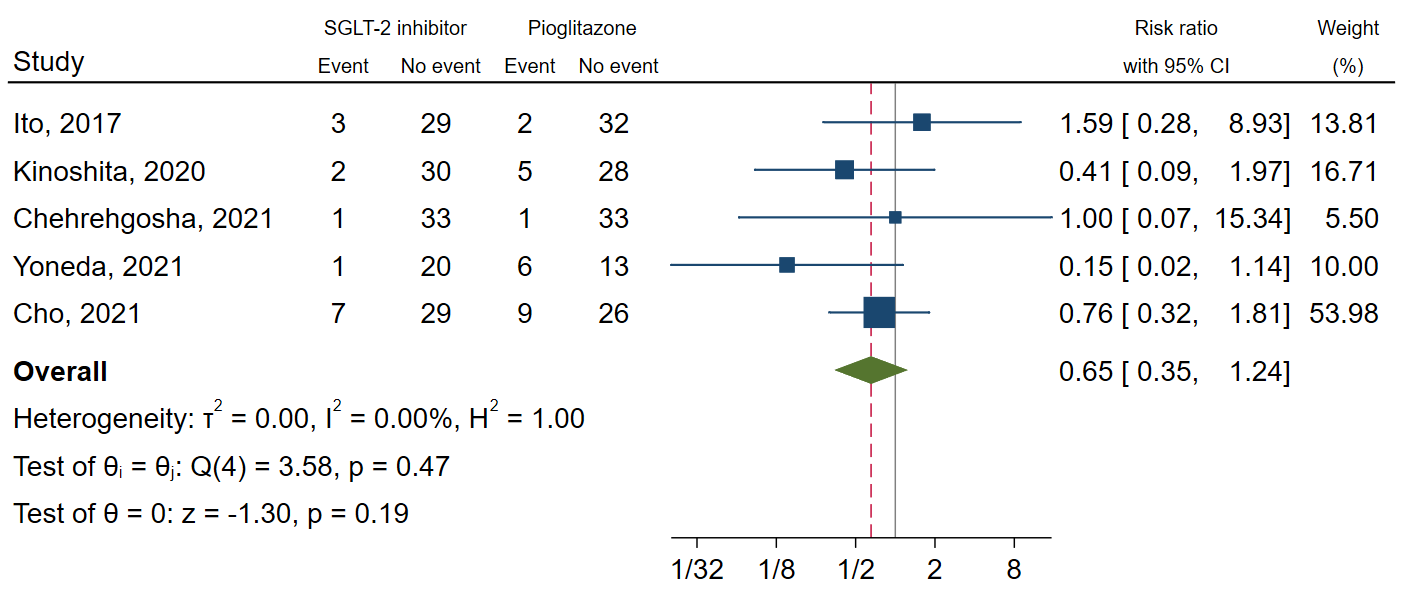


**Supplementary figure S2:** SUCRA graph of each outcome

1. **Intrahepatic steatosis evaluated by MRI**

**
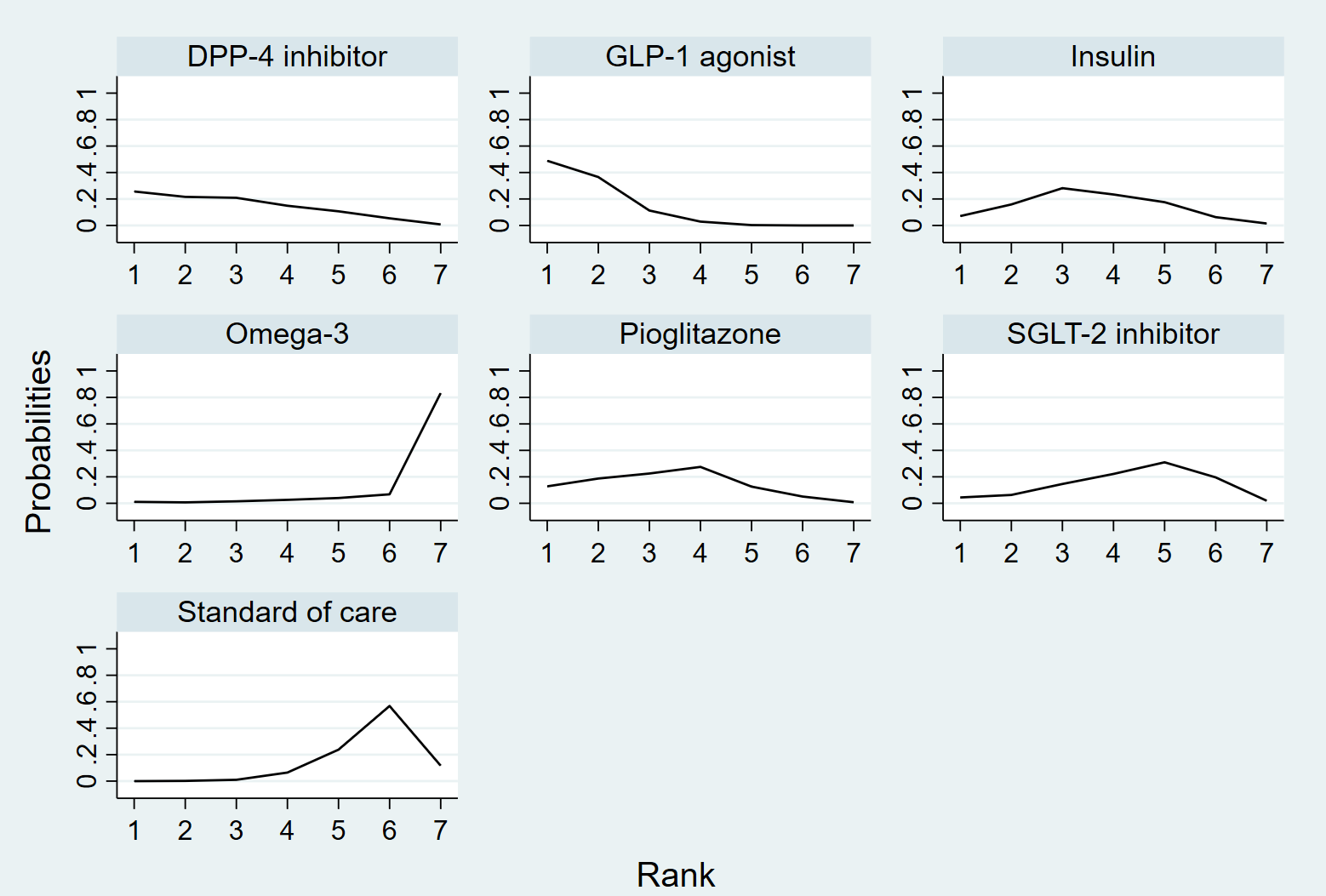
**

1. **AST**


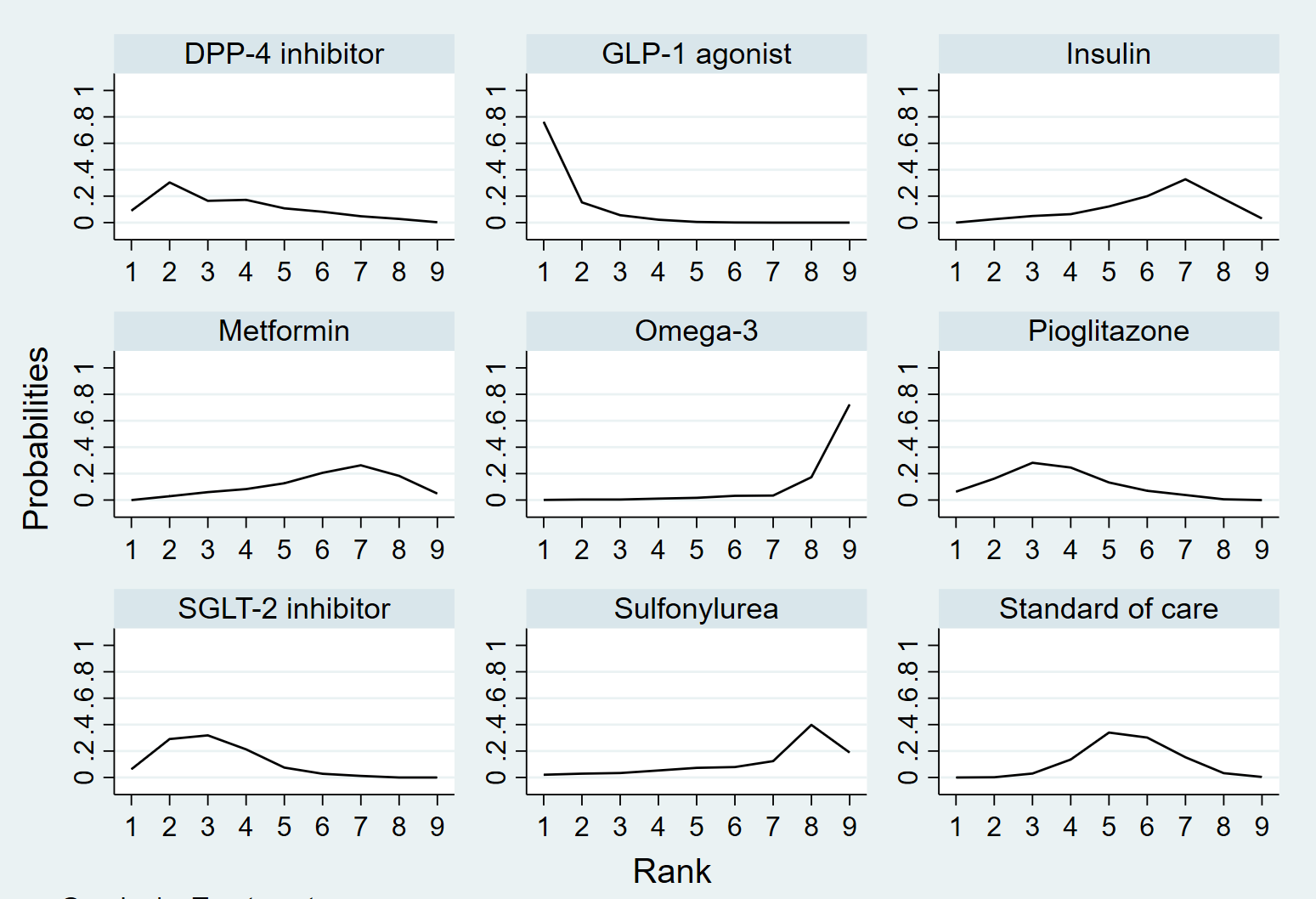


1. **ALT**


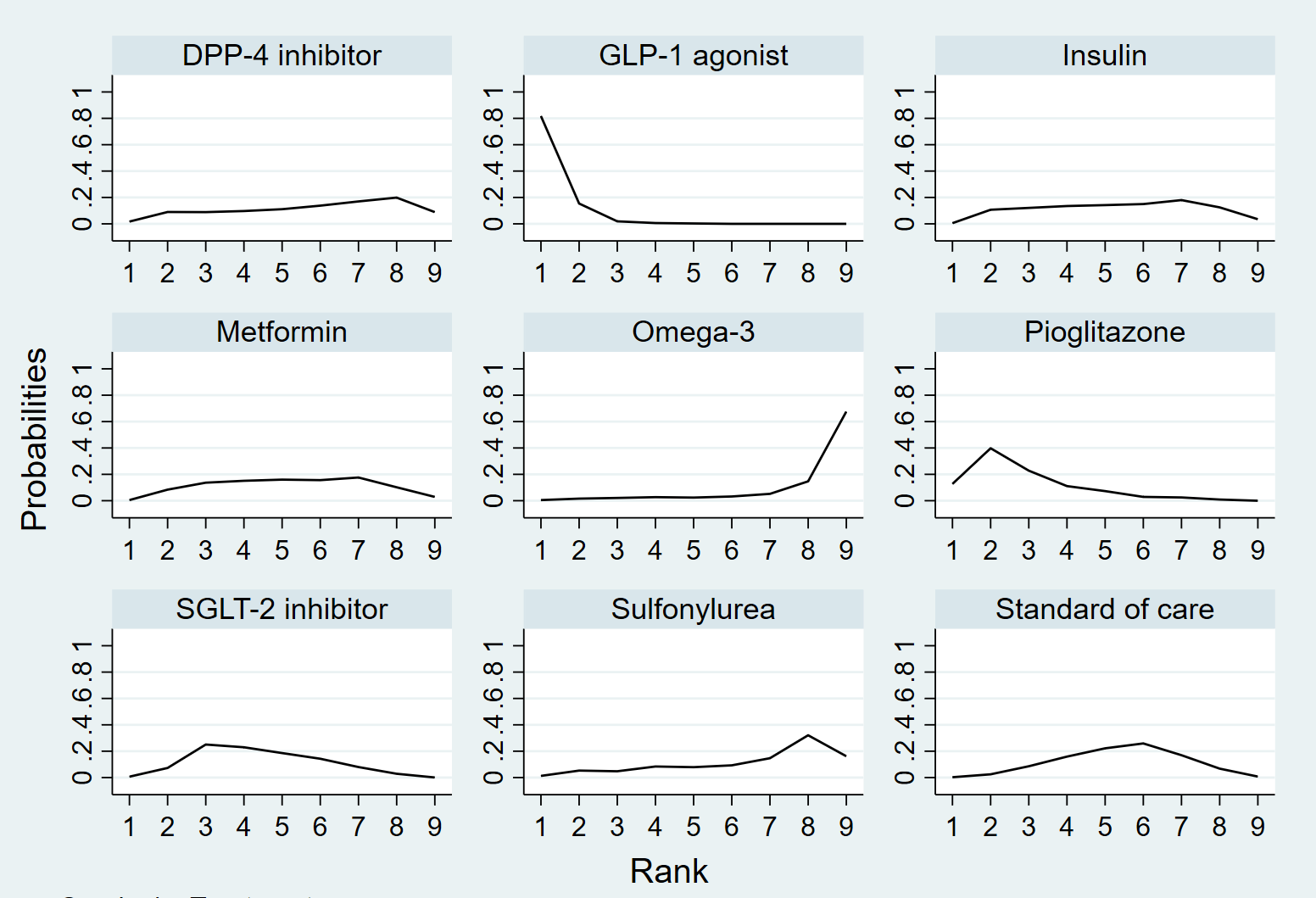


1. **GGT**

**
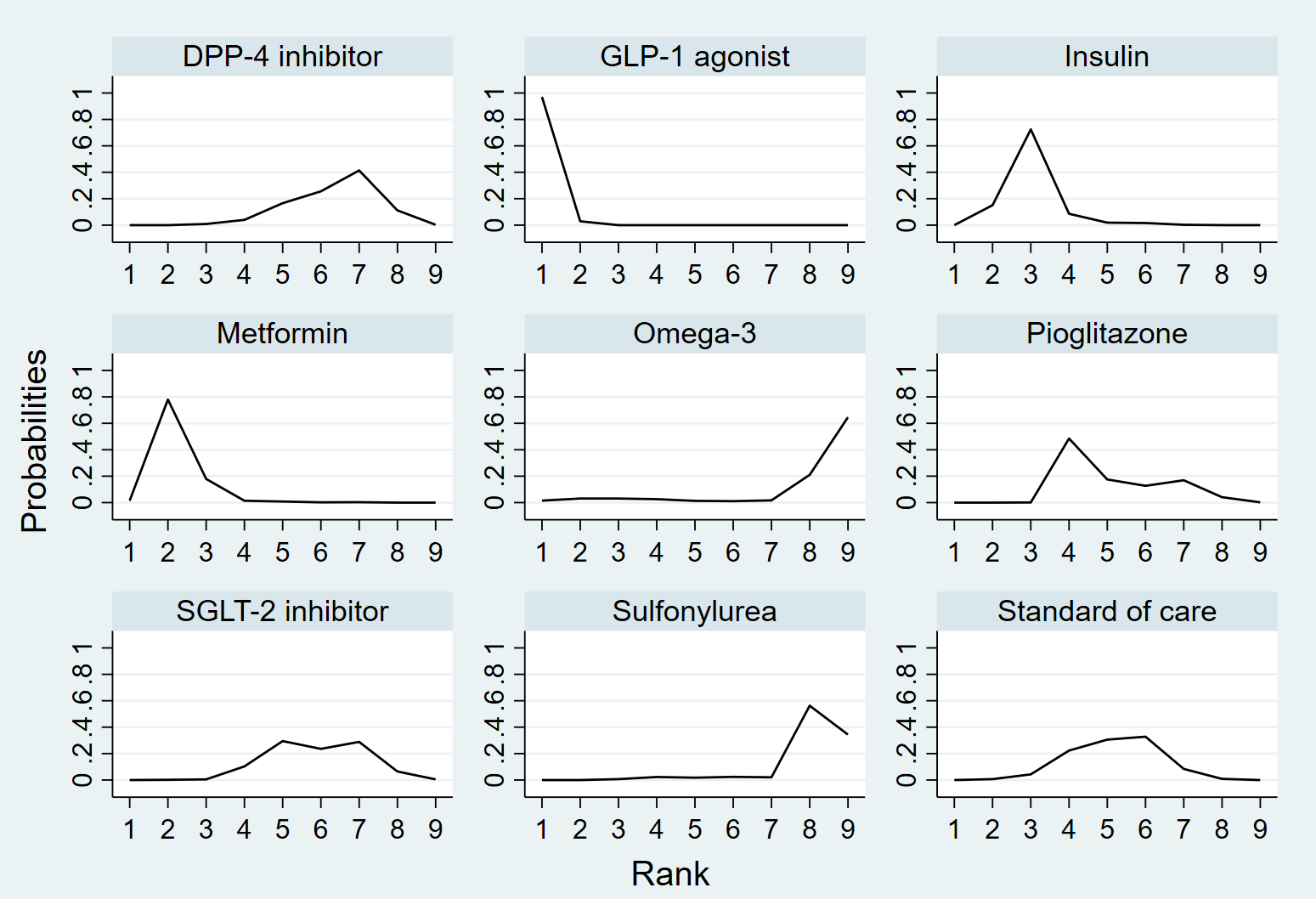
**

1. **BMI**


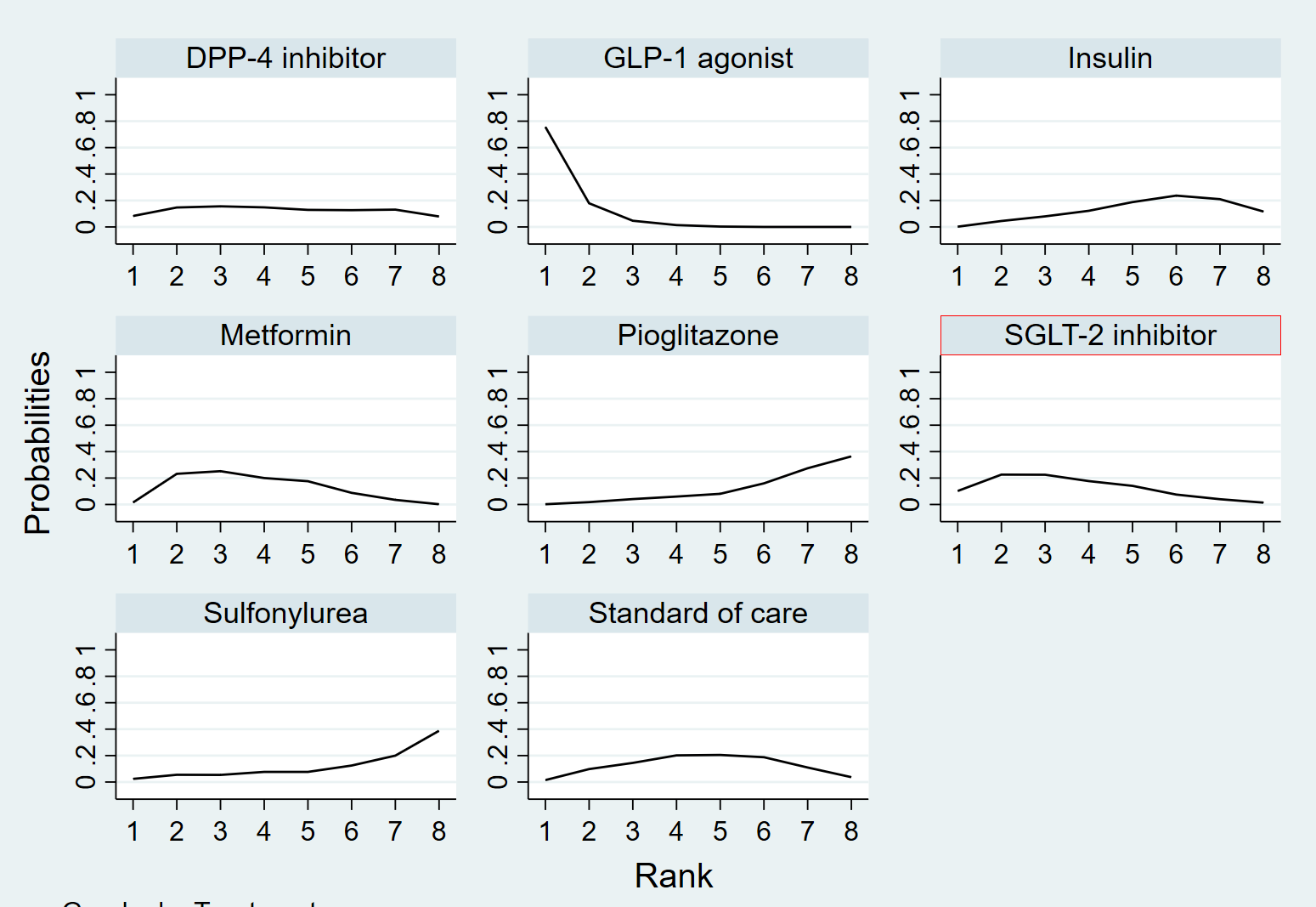


1. **Any adverse event**


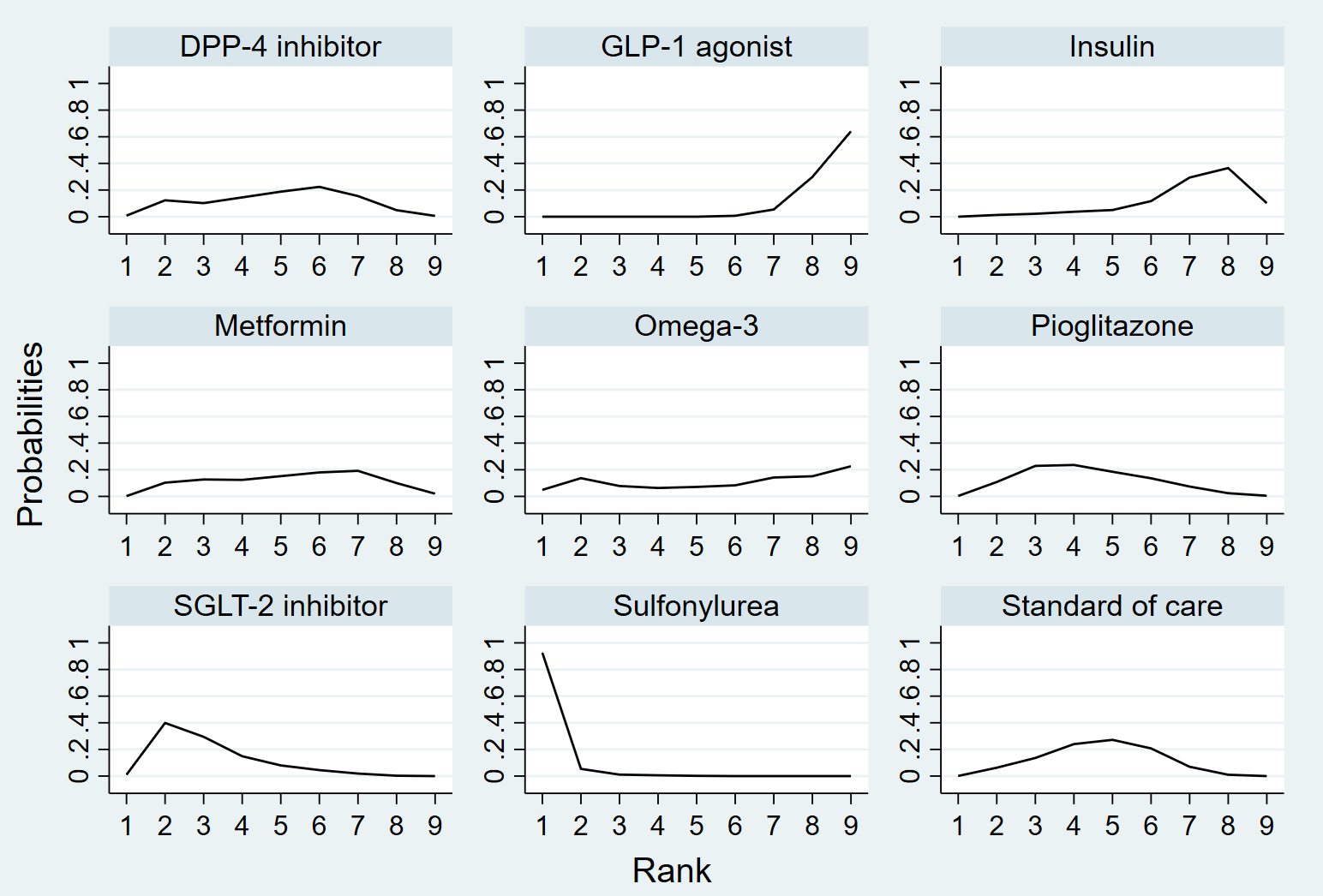


**Supplementary Figure S3:** Risk of bias assessment for studies included.


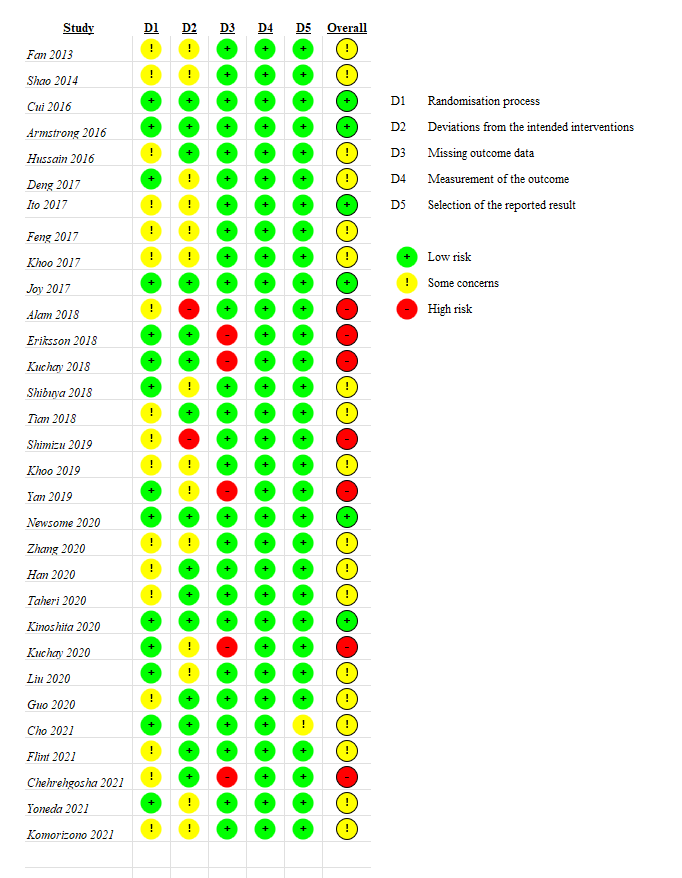


**Supplementary Figure S4:** Comparison-adjusted funnel plot

1. **Comparison-adjusted funnel plot of AST**


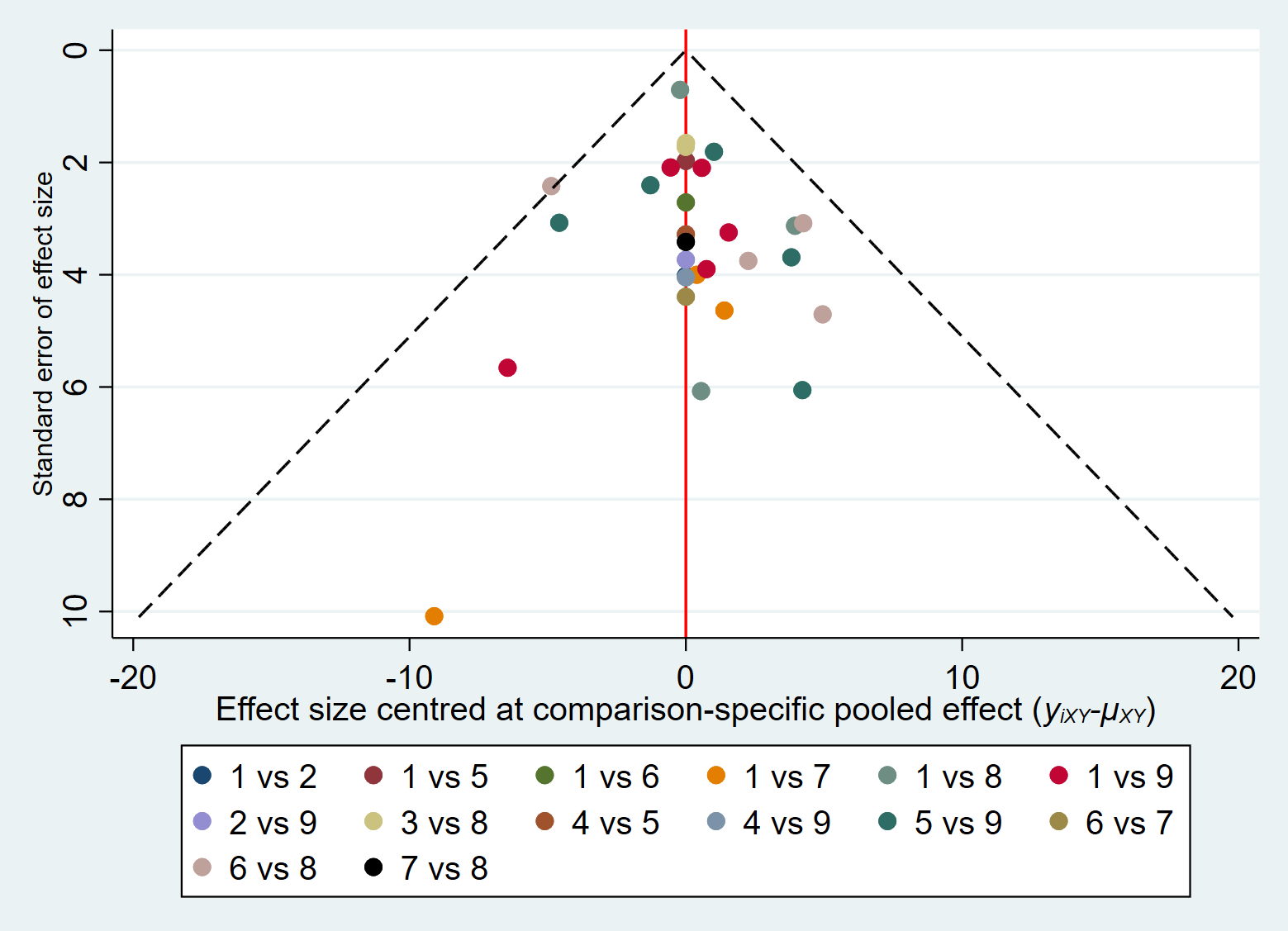


1 = standard of care; 2 = omega-3; 3 = metformin; 4 = sulfonylurea; 5 = pioglitazone; 6 = insulin; 7 = DPP-4 inhibitor; 8 = GLP-1 agonist; 9 = SGLT-2 inhibitor.

1. **Comparison-adjusted funnel plot of ALT**


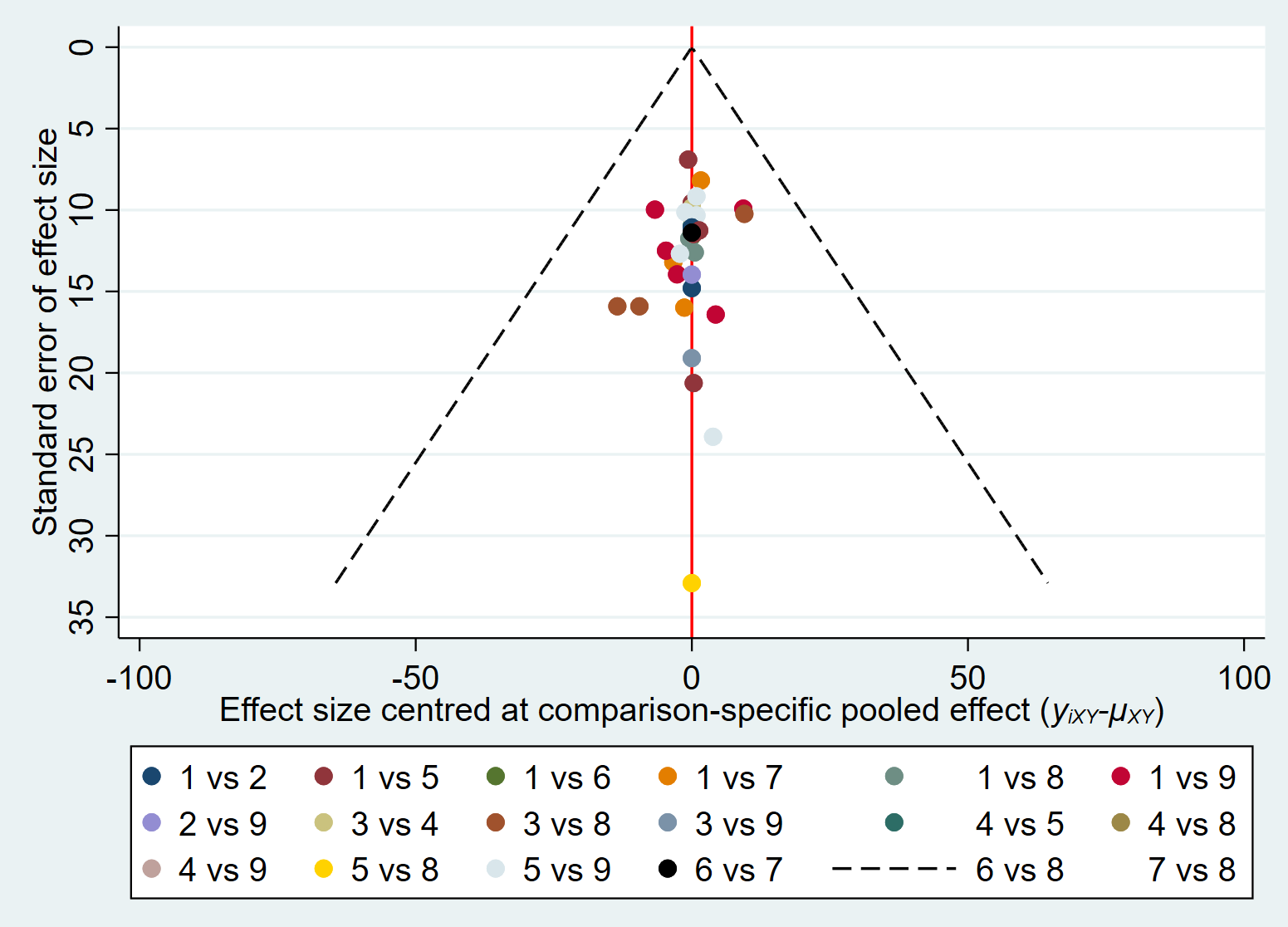


1 = standard of care; 2 = omega-3; 3 = metformin; 4 = sulfonylurea; 5 = pioglitazone; 6 = insulin; 7 = DPP-4 inhibitor; 8 = GLP-1 agonist; 9 = SGLT-2 inhibitor.

1. **Comparison-adjusted funnel plot of GGT**

**
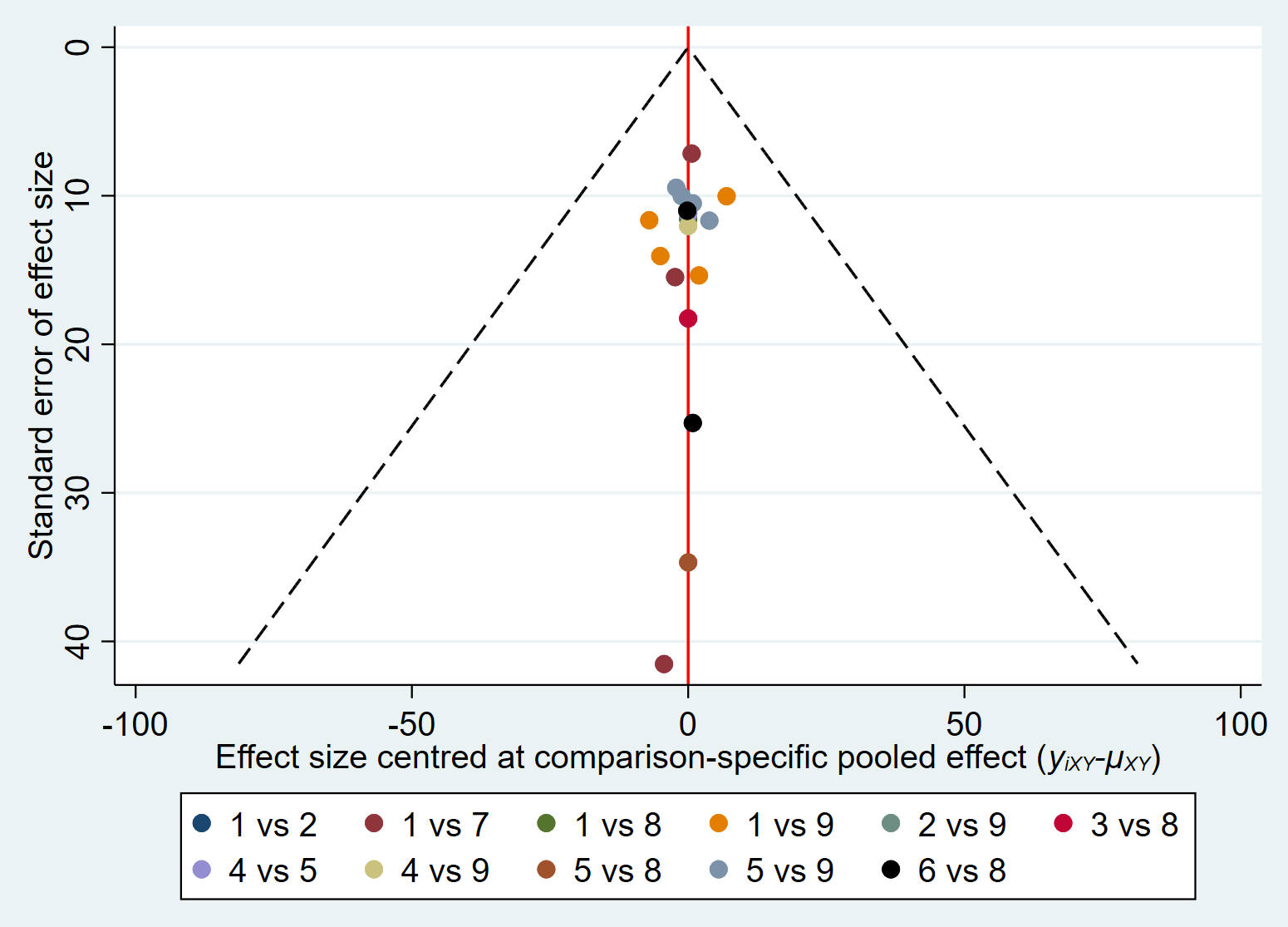
**

1 = standard of care; 2= omega-3; 3 = metformin; 4 = sulfonylurea; 5 = pioglitazone; 6 = insulin; 7 = DPP-4 inhibitor; 8 = GLP-1 agonist; 9 = SGLT-2 inhibitor.

1. **Comparison-adjusted funnel plot of intrahepatic steatosis by MRI**

**
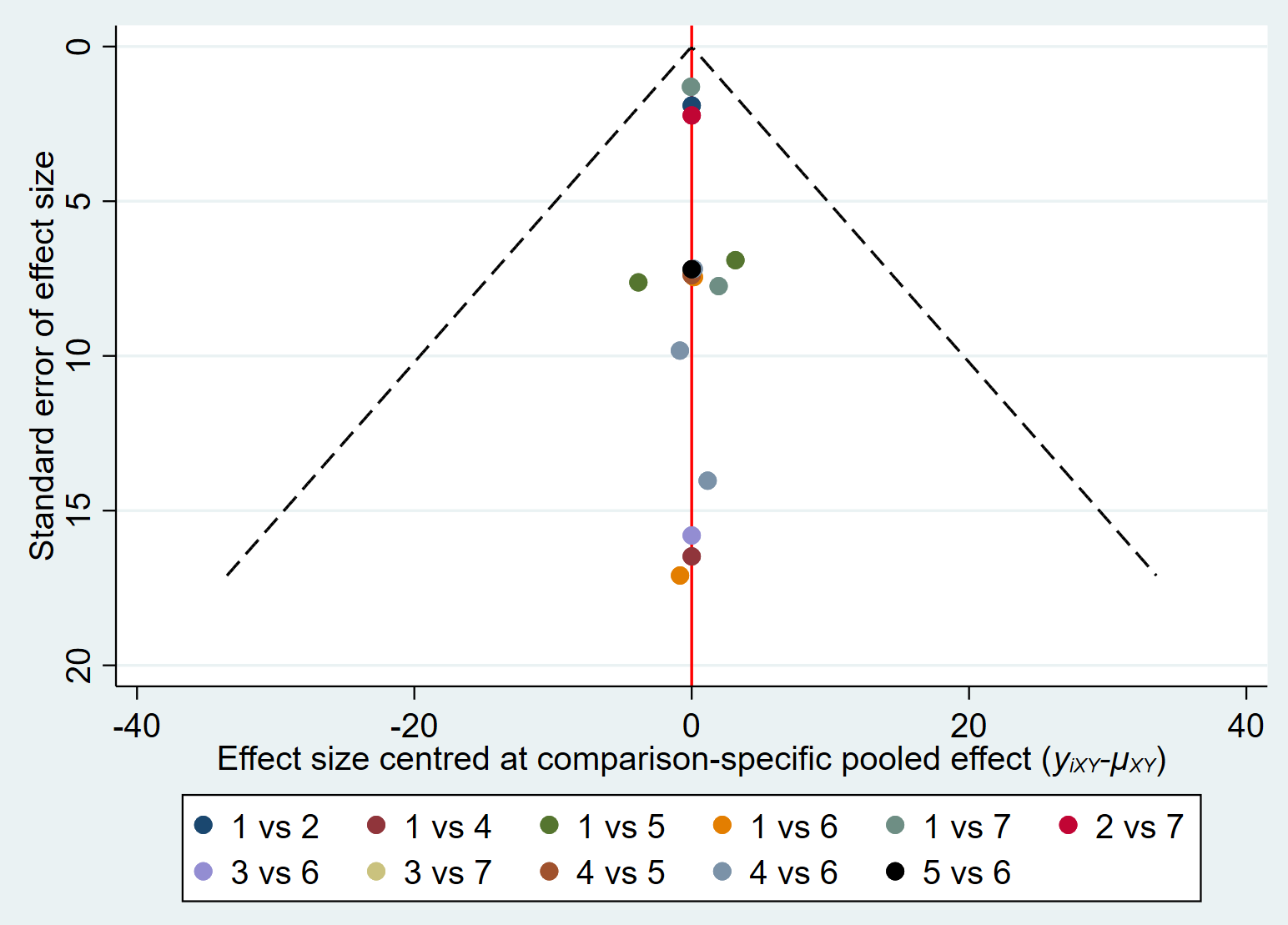
**

1 = standard of care; 2= omega-3; 3 = pioglitazone; 4 = insulin; 5 = DPP-4 inhibitor; 6 = GLP-1 agonist; 7 = SGLT-2 inhibitor.

1. **Comparison-adjusted funnel plot of BMI**


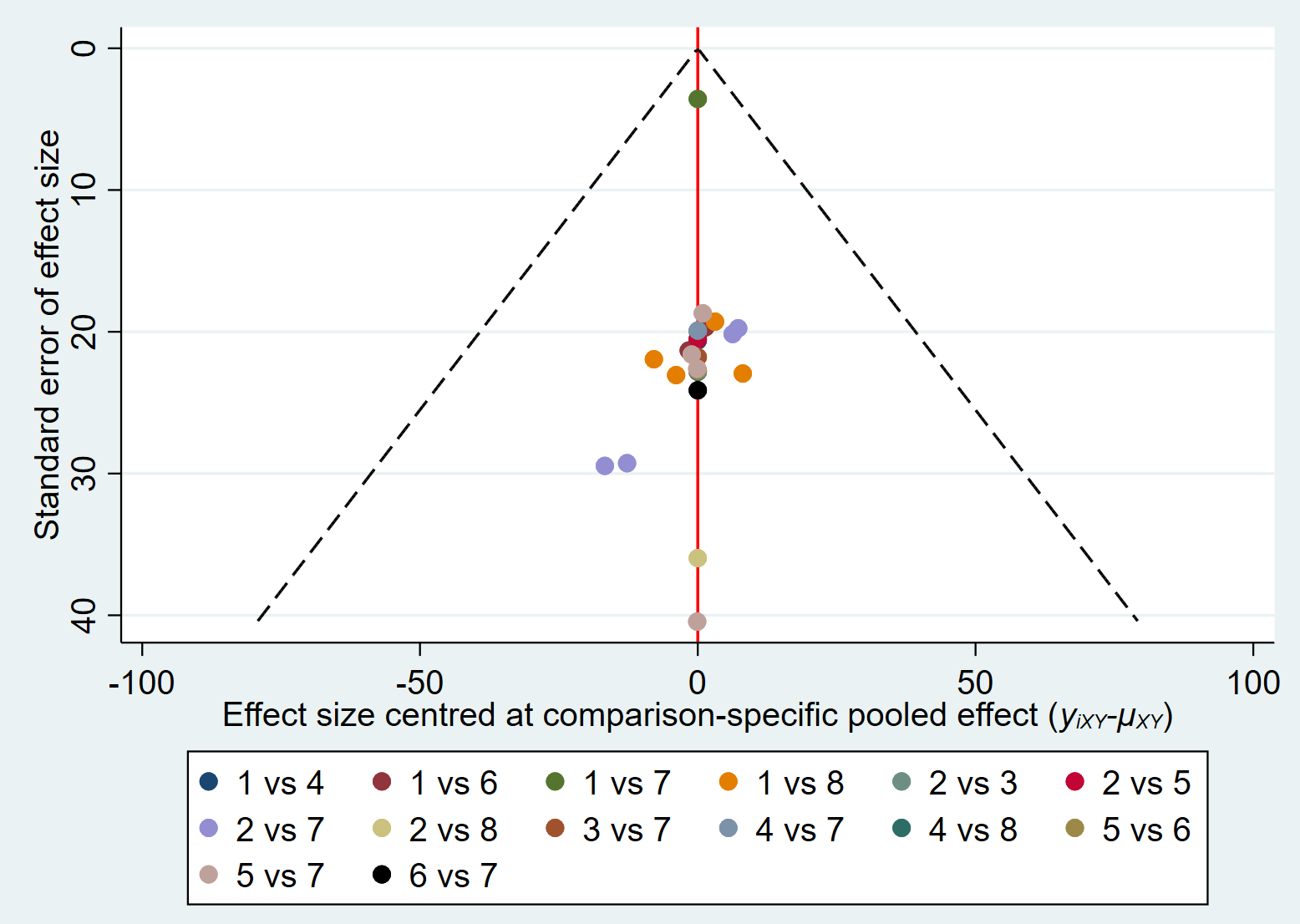


1 = standard of care; 2 = metformin; 3 = sulfonylurea; 4 = pioglitazone; 5 = insulin; 6 = DPP-4 inhibitor; 7 = GLP-1 agonist; 8 = SGLT-2 inhibitor.

1. **Comparison-adjusted funnel plot of any adverse event**

**
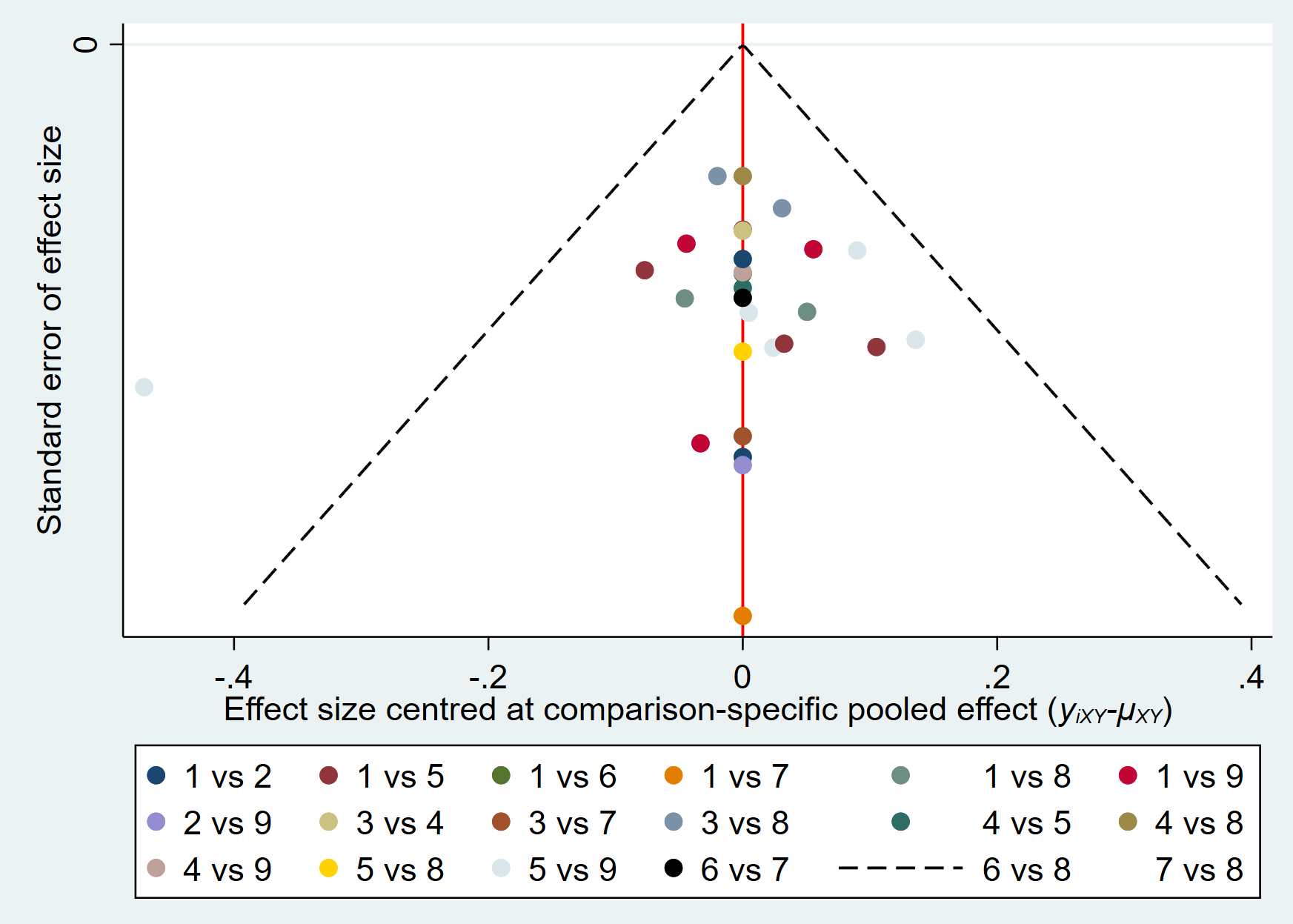
**

1 = standard of care; 2= omega-3; 3 = metformin; 4 = sulfonylurea; 5 = pioglitazone; 6 = insulin; 7 = DPP-4 inhibitor; 8 = GLP-1 agonist; 9 = SGLT-2 inhibitor.
